# Serum-derived extracellular vesicles (EVs) impact on vascular remodeling and prevent muscle damage in acute hind limb ischemia

**DOI:** 10.1038/s41598-017-08250-0

**Published:** 2017-08-15

**Authors:** Claudia Cavallari, Andrea Ranghino, Marta Tapparo, Massimo Cedrino, Federico Figliolini, Cristina Grange, Valentina Giannachi, Paolo Garneri, Maria Chiara Deregibus, Federica Collino, Pietro Rispoli, Giovanni Camussi, Maria Felice Brizzi

**Affiliations:** 1 Department of Medical Sciences, 2i3T Scarl, University of Turin, Turin, Italy; 2 Department of Surgical Sciences, University of Turin, Turin, Italy

## Abstract

Serum is an abundant and accessible source of circulating extracellular vesicles (EVs). Serum-EV (sEV) pro-angiogenic capability and mechanisms are herein analyzed using an *in vitro* assay which predicts sEV angiogenic potential *in vivo*. Effective sEVs (e-sEVs) also improved vascular remodeling and prevented muscle damage in a mouse model of acute hind limb ischemia. e-sEV angiogenic proteomic and transcriptomic analyses show a positive correlation with matrix-metalloproteinase activation and extracellular matrix organization, cytokine and chemokine signaling pathways, Insulin-like Growth Factor and platelet pathways, and Vascular Endothelial Growth Factor signaling. A discrete gene signature, which highlights differences in e-sEV and ineffective-EV biological activity, was identified using gene ontology (GO) functional analysis. An enrichment of genes associated with the Transforming Growth Factor beta 1 (TGFβ1) signaling cascade is associated with e-sEV administration but not with ineffective-EVs. Chromatin immunoprecipitation analysis on the *inhibitor of DNA binding I* (ID1) promoter region, and the knock-down of *small mother against decapentaplegic* (SMAD)1–5 proteins confirmed GO functional analyses. This study demonstrates sEV pro-angiogenic activity, validates a simple, sEV pro-angiogenic assay which predicts their biological activity *in vivo*, and identifies the TGFβ1 cascade as a relevant mediator. We propose serum as a readily available source of EVs for therapeutic purposes.

## Introduction

Critical limb ischemia (CLI), the most severe consequence of peripheral artery disease, is a rife condition caused by acute occlusion of the peripheral arteries^1^. Although surgical or endovascular intervention are still the standard therapy for improving blood flow^2^, most patients complain of persistent or recurring symptoms even after successful revascularization^3^. This implies that new therapeutic options for patients complying these symptoms are very much still a major unmet need.

The formation of newly born capillary blood vessels, neovascularization, is a crucial event in rescuing tissue after ischemia. A vast array of pre-clinical and clinical data indicates that stem cells can contribute to the healing processes by improving vascularization^4, 5^. However, the transient detection of stem cells at the site of tissue damage has suggested that paracrine mechanisms may contribute to stem cell action^6, 7^. “Exosomes”, vesicles derived from the endosomal membrane compartment by exocytosis, and “ectosomes/microvesicles”, vesicles generated by the budding of cell plasma membranes^8^ are included among such paracrine mechanisms. Due to the overlap in the characteristics and biological activity of exosomes and ectosomes/microvesicles^9^, it has recently been suggested that a more inclusive term, “extracellular vesicles” (EVs), be used. EVs are powerful cell-to-cell communication mediators in both physiological and pathological conditions^10^. Recent studies have provided evidence that the transfer of EV cargo can dictate recipient cell fate^11, 12^. Indeed, the transfer of EV cargo, including nucleic acids and proteins, into recipient cells is the most relevant mechanism of EV action^13, 14^. The most studied EVs are currently those released by stem cells, thanks to their ability to mimic the effects of the cell of origin^14^. A range of different phenotypic changes, depending on the pathological setting, have been described in target cells^15^. Transcriptomic and proteomic analyses have often been performed in an attempt to try to characterize EV mechanism of action^11, 16^. In particular, a robust pro-angiogenic profile has been described in EVs recovered from mesenchymal stem cells derived from bone marrow or adipose tissues^5, 17^. These observations have hinted at the potential impact of EVs in clinical settings, including ischemia-related diseases^18, 19^. The relevance of EVs in physiological and pathological angiogenesis and their mechanisms of action have been extensively reviewed by Kholia *et al*.^20^.

EVs that have been physiologically released from several cell types can be detected in biological fluids, including blood^21–24^. The EVs present in blood could therefore be an important resource for regenerative medicine. Indeed, plasma EVs have been reported to provide a cardioprotective effect against ischemia-reperfusion injury^25^. Experiments into the depletion of EVs in fetal bovine serum have displayed an impaired ability to induce muscle cell proliferation and differentiation^26^, suggesting that serum EVs (sEVs) possess defined biological properties.

The aim of the present study was to mechanistically investigate the angiogenic potential of EVs that had been purified from the sera of healthy donors (sEVs). In particular, the possible therapeutic impact of sEVs in inducing angiogenesis and improving the reperfusion of acute hind limb ischemia has been investigated. Moreover, an mRNA-angiogenesis microarray has been performed on endothelial cells (ECs) recovered from capillary-like structures formed in response to sEVs in order to dissect their mechanism(s) of action.

## Results

### Characterization and functional activity of sEVs

All EV samples were analyzed for their surface antigens via Guava FACS analysis. As expected, it was demonstrated that sEVs are heterogeneous due to their differing cells of origin (platelets, endothelial cells, monocytes/leukocytes) (Supplementary Table S1). The sEV expression of cell adhesion markers was also detected (Supplementary Table S1). Nanosight analyses did not bring to light any significant differences in sEV number and size among the samples. The number of sEVs recovered from a bag of 110 ml was around 6.16 × 10^8^ particles/ml (Supplementary Fig. S1), while sEV diameter was approximately 210 nm with a modal particle size of 183 nm (Supplementary Fig. S1). Starting from 2 × 10^11^ of total particles, the recovery of sEV RNA and protein was similar in all samples analyzed (n = 18), and ranged from 300 to 500 ng of RNA and 1 to 1.2 µg of protein (Supplementary Fig. S1). To avoid possible contamination of non-sEV-associated mRNAs and proteins, floating density gradient was used to separate sEVs^27, 28^. The expression of the CD63 exosomal marker was also detected, using Western Blot (WB) analysis, in the 30% iodixanol floating fraction (see Methods) (Supplementary Fig. S2).

In order to try and predict the pro-angiogenic activity of sEVs, an *in vitro* assay was performed using VEGF as the internal control. Effective sEVs (e-sEVs) were considered to be those demonstrating almost 50% of VEGF proangiogenic activity when analyzed for their ability to induce ECs proliferation and tube-like structure formation. As shown in Table 1, 14 out of 18 sEV samples displayed such a feature.

**Table 1:** Proangiogenic *in vitro* effects of different sEV samples.

sEVs	Angiogenesis assay %	Proliferation assay %	Average %	Results
1	42.8 ± 1.4	62.5 ± 2.3	52.6 ± 1.9	e-sEV
2	54.4 ± 3.9	56.4 ± 3.1	55.4 ± 3.5	e-sEV
3	31.9 ± 1.4	89.5 ± 0.5	60.7 ± 1.0	e-sEV
4	53.3 ± 0.3	64.1 ± 1.1	58.7 ± 0.7	e-sEV
5	68.7 ± 2.6	55.5 ± 3.1	62.1 ± 2.9	e-sEV
6	43.8 ± 0.9	77.6 ± 4.0	60.7 ± 2.5	e-sEV
7	88.5 ± 4.2	66.2 ± 3.3	77.4 ± 3.8	e-sEV
8	65.5 ± 0.6	44.4 ± 0.5	54.9 ± 0.6	e-sEV
9	56.5 ± 4.5	50.1 ± 3.9	53.3 ± 4.2	e-sEV
10	31.5 ± 1.1	99.8 ± 5.6	65.7 ± 3.4	e-sEV
11	35.2 ± 8.3	8.5 ± 4.1	21.9 ± 6.2	i-sEV
12	99.2 ± 0.5	32.2 ± 3.4	65.7 ± 2.0	e-sEV
13	28.8 ± 3.2	24.5 ± 2.3	26.7 ± 2.8	i-sEV
14	41.7 ± 6.6	68.6 ± 4.5	55.2 ± 5.6	e-sEV
15	50 ± 3.8	55 ± 4.9	52.5 ± 4.4	e-sEV
16	8.3 ± 1.3	19.1 ± 4.0	13.7 ± 2.5	i-sEV
17	66.7 ± 4.5	41.1 ± 4.2	53.9 ± 6.6	e-sEV
18	15.1 ± 1.5	3.6 ± 0.6	9.5 ± 2.6	i-sEV

18 sEV samples evaluated by *in vitro* angiogenesis assay and *in vitro* proliferation assay on ECs. The average represents the mean value of results of both assays ± SD (see Methods). sEVs with an average value exceeding 50% have been considered effective sEVs (e-sEVs) otherwise they are ineffective (i-sEVs).

### sEVs induce *in vitro* and *in vivo* angiogenesis

**Figure 1:**
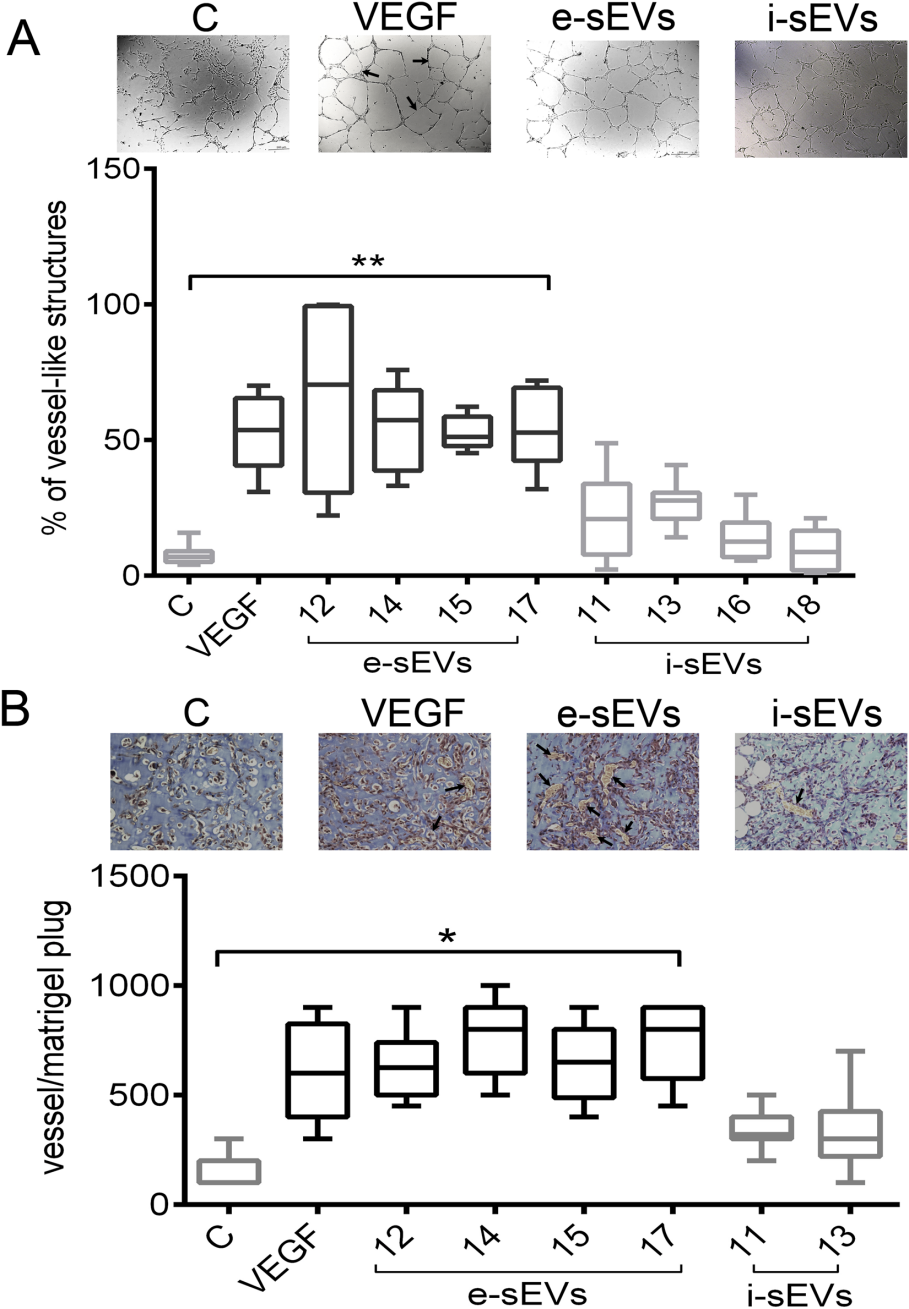
*In vitro* and *in vivo* angiogenesis in response to sEVs. (**A**) *In vitro* quantitative analysis of the % of vessel-like structures formed (tubulogenesis assay) in response to the indicated stimuli. Untreated ECs was the negative control, VEGF (10 ng/ml) was the positive control, while e-sEVs (samples # 12, 14, 15, 17) and i-sEVs (samples # 11, 13, 16, 18) were the stimuli. (Original magnification: ×200; scale bar: 500 μm). (**B**) *In vivo* quantitative analysis of vessels counted in 10 sections of matrigel for each experimental condition. Data represent the mean value of untreated (**C**) (n = 6) or treated mice with: VEGF (n = 6), e-sEVs (samples # 12, 14, 15, 17) (n = 6 each sample) and i-sEVs (samples # 11, 13) (n = 6 each sample). ECs (red), erythrocytes (yellow) and matrigel (light blue) staining in matrigel plugs. Images above the graphs are representative of each condition. (Original magnification: ×20). Arrows indicate vessels. *p < 0.05; **p < 0.01, (One-way ANOVA followed by Tukey’s post hoc test). (Original magnification: ×200; scale bar: 12 μm).

A comparison of *in vitro* and *in vivo* experiments was performed in order to validate the predicted sEV pro-angiogenic activity. The *in vitro* tridimensional angiogenic assay was used to analyze four distinct samples for both e-sEVs and ineffective s-EVs (i-sEVs). As shown in Fig. 1A, e-sEVs were able to induce tube-like structure formation *in vitro*, unlike i-sEVs. In order to verify these results *in vivo*, selected sEVs were used in SCID mice. ECs treated with effective and ineffective sEVs (5 × 10^4^ sEV/cell) and mixed with Matrigel were injected subcutaneously into SCID mice for this purpose. Matrigel plugs were removed and analyzed after one week. As shown in Fig. 1B, only e-sEVs displayed pro- angiogenic activity *in vivo*. These data indicate that our *in vitro* assays were able to predict sEVs pro-angiogenetic capability.

### sEVs improve reperfusion recovery and prevent muscle damage after ischemia

**Figure 2:**
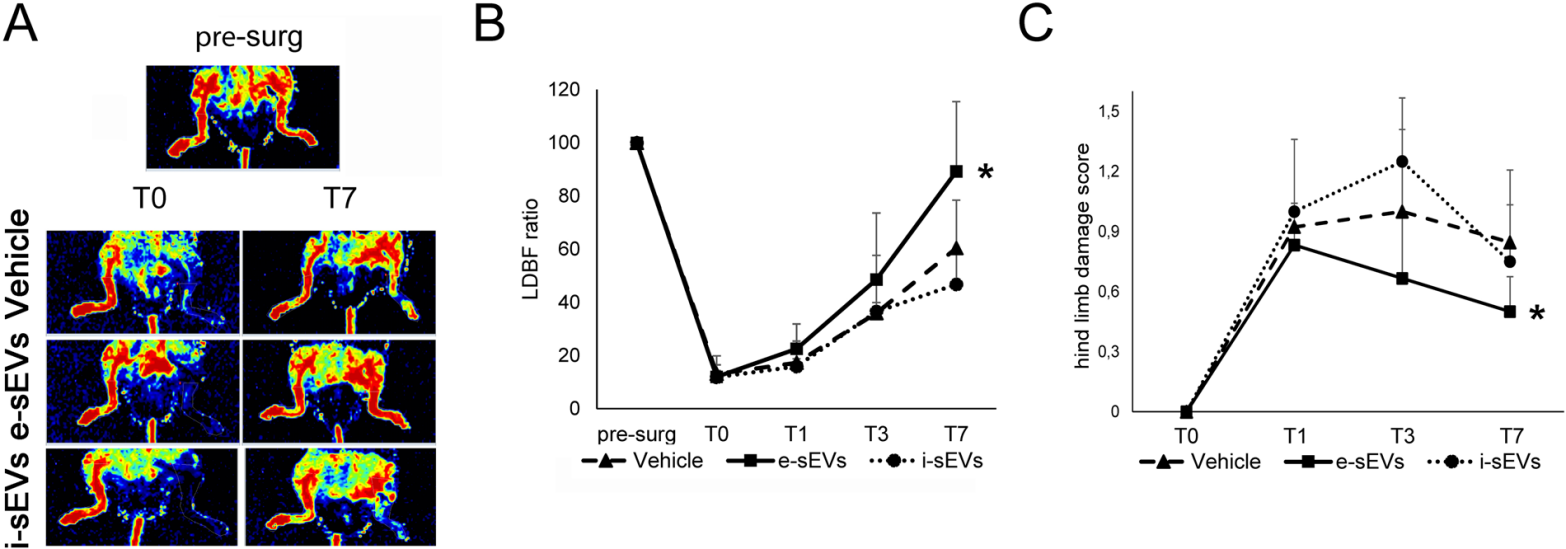
e-sEVs increase blood perfusion and reduced damage in ischemic hind limbs. (**A**) Representative images of Laser Doppler Blood Flow (LDBF) obtained prior (pre-surg) and immediately after intervention (T0) and at day 7 after surgery (T7) of mice treated with Vehicle, with e-sEVs and i-sEVs. (**B**) Quantitative analysis of blood perfusion measured by LDBF. Data are expressed as mean ± SD; *p  < 0.05 (One-way ANOVA - Newman-Keuls Multiple Comparison Test) (n = 10). (**C**) Foot damage score was evaluated for the indicated times as reported in Methods. Data are expressed as mean ± SEM, n = 10 (*p < 0.0*5* ischemic limb of e-sEVs mice vs ischemic limb of vehicle and i-sEVs mice).

**Figure 3:**
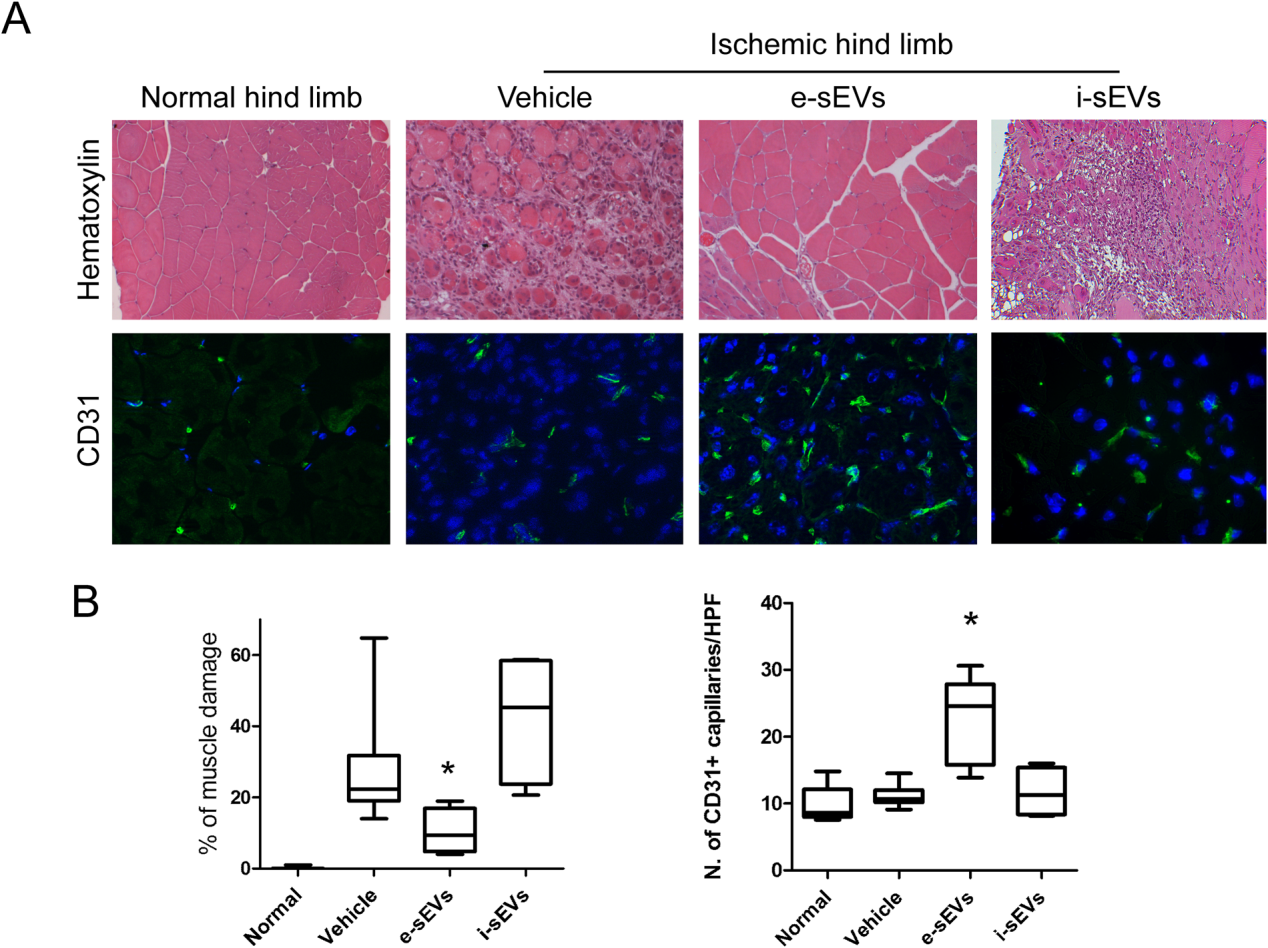
e-sEVs increase capillary density and protect muscle from ischemic damage. (**A**) Representative hematoxylin-eosin images of gastrocnemius muscles of non-ischemic (Normal hind limb) and ischemic hind limb of Vehicle, e-sEVs and i-sEVs treated animals (Original Magnification: ×200; scale bar: 50 µm), (upper panels). Representative immunofluorescence images (confocal microscopy) of capillary density staining. An anti-CD31 mAb was used to analyze gastrocnemius muscles of non-ischemic (Normal hind limb) and ischemic hind limbs as indicated (Original Magnification: ×400; scale bar: 25 µm) (lower panels). (**B**) Quantitative analysis of muscle damage areas in hematoxilin-eosin stained gastrocnemius muscles of normal and ischemic hind limbs at day 7 after surgery. e-sEVs significantly reduce muscle damage compared to Vehicle. Data are expressed as mean ± SEM; *p  < 0.05 e-sEVs vs Vehicle, (One-way ANOVA - Newman-Keuls Multiple Comparison Test), (left panel) (n = 10). Quantitative analysis of capillary density of normal and ischemic hind limbs at day 7 after surgery indicates that e-sEVs significantly increase capillary density compared to Vehicle. Data are expressed as mean ± SEM; *p < 0.05, e-sEVs vs Vehicle, (One-Way ANOVA - Newman-Keuls Multiple Comparison Test), (right panel).

**Figure 4:**
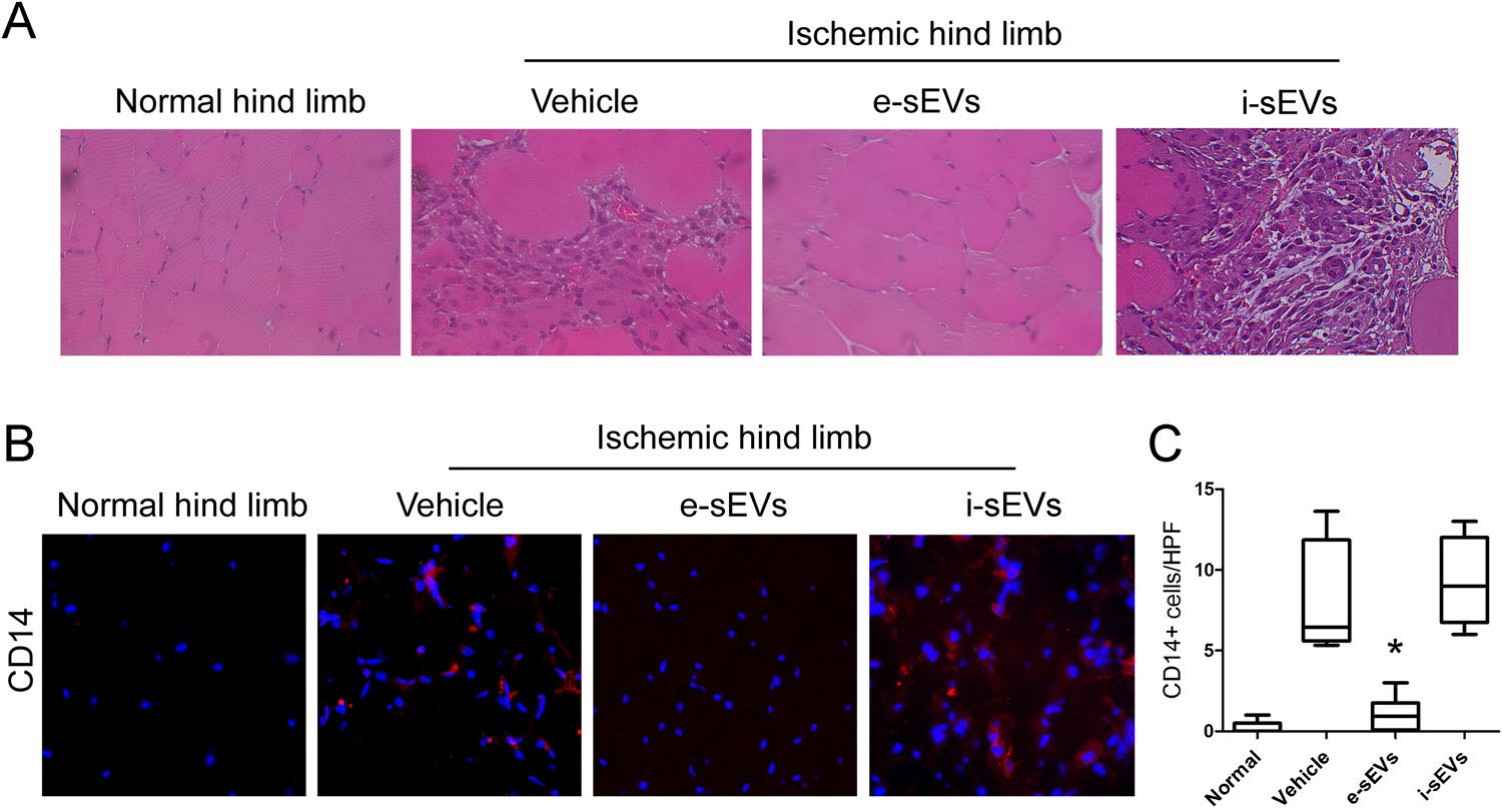
Inflammatory infiltrate in ischemic hind limb. (**A**) Representative hematoxylin-eosin images of gastrocnemius muscles of non-ischemic (Normal hind limb) and ischemic hind limbs of Vehicle, e-sEVs and i-sEVs treated animals (Original Magnification: ×400; scale bar: 25 µm), showing mononuclear and polymorphonuclear cells. (**B**) Representative immunofluorescence images of gastrocnemius muscles, stained with an anti CD14 antibody (Original Magnification: ×400; scale bar: 25 µm). (**C**) Quantitative analysis of muscle damage areas in hematoxylin-eosin stained gastrocnemius muscles of the normal and ischemic hind limbs at day 7 after surgery. e-sEVs significantly reduce the number of inflammatory cells compared to the control (vehicle). Data are expressed as mean ± SEM; *p < 0.05, e-sEVs vs Vehicle, (One-Way ANOVA - Newman-Keuls Multiple Comparison Test) (n = 6).

e-sEVs were also evaluated in a murine model of acute ischemic hind limbs that mimics CLI in humans. A total amount of 2 × 10^10^ e-sEVs was administrated after surgery (see Methods). Different routes of i-sEV and e-sEV administration were assayed in order to evaluate their therapeutic potential (data not shown). The best response, was obtained as follows: intravenous injection immediately after surgery (T0) and intramuscular at T1 and T2. This approach was used throughout the study. Blood perfusion in the ischemic hind limb, as evaluated by Laser Doppler Perfusion imaging immediately after surgery, demonstrated that the ratio between the ischemic and non-ischemic hind limbs decreased in all groups (0.13 ± 0.06 control group; 0.12 ± 0.04 in e-sEV group; and 0.11 ± 0.02 in i-sEV group) (Fig. 2). With the limitation of this approach, which does not allow quantification of deep vascular districts^29, 30^, we found that e-sEVs, but not i-sEVs and vehicle-treated animals, displayed a significant increased hind limb perfusion ratio at day 7 after surgery (e-sEVs 0.89 ± 0.26 vs control 0.6 ± 0.18 and vs i-sEVs 0.46 ± 0.13, p < 0.05) (Fig. 2B). Functional score applied to all groups demonstrated that the damage was also significantly higher in vehicle and i-sEV-treated groups than in the e-sEV-treated group (Fig. 2C)^19, 31^. Consistently, capillary density in the ischemic hind limbs was significantly increased in e-sEV-group of animals (vessel number/HPF: e-sEVs 22.4 ± 5.5 vs control 11.2 ± 1.8 and vs i-sEVs 11.68 ± 3.7, p < 0.05) (Fig. 3). The analysis of the gastrocnemius muscles of either i-sEVs and vehicle-treated animals revealed the presence of focal areas of necrosis and inflammation (Figs 3 and 4). CD14 positive cells and polymorph-nuclear cells were also detected in vehicle and i-sEVs-treated mice (Fig. 4). The necrosis and infiltrate of inflammatory cells were almost completely prevented by e-sEV administration (Fig. 4). Finally, even if at day 7 in vehicle and i-sEV-treated animals the capillary density was improved in respect to day 1 (not shown), it was not sufficient to prevent muscle damage.

### e-sEV-cargo analysis

**Figure 5:**
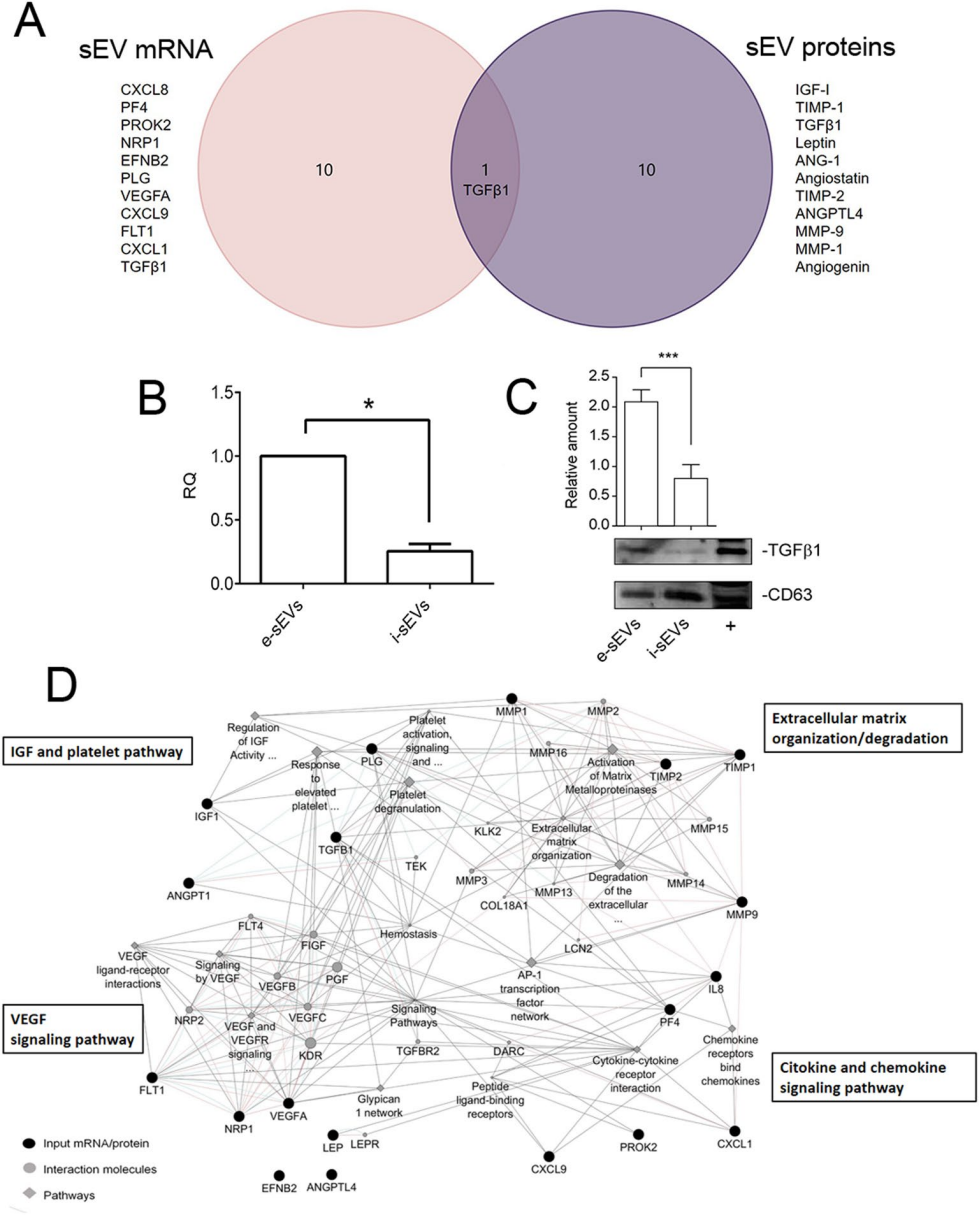
e-sEV angiogenic mRNA and protein content. (**A**) Venn diagram to show the first 11 angiogenic mRNAs and proteins expressed by e-sEVs. TGFβ1 was commonly detected as mRNAs and proteins. Data were analyzed by Funrich V3 tool. (**B**) RT-PCR was used to detect TGFβ1 mRNA in e-sEV cargo. RT-PCR data are expressed as relative quantification (RQ) ± SD (*p < 0.05) (n = 4). (**C**) e-sEV were analyzed by Western Blot analysis for TGFβ1 content and normalized to CD63 (*n* = 4) (***p < 0.001). Mesangial cells were used as positive control (+). (**D**) Network analysis of pathways positively correlated with e-sEV transcripts and proteins. Pathways positively correlated are reported in the boxes. Data were obtained by GeneMANIA analysis performed on Cytoscape.

The above results led us to investigate how angiogenic-related mRNAs and proteins contribute to e-sEV biological activity. According to an mRNA and protein microarray analysis (Supplementary Table S2), we selected the first 11 angiogenic mRNAs and proteins expressed by e-sEVs (Fig. 5A). TGFβ1 was commonly detected when mRNA and protein in e-sEVs was compared. The presence of TGFβ1 was also validated by real time-PCR (RT-PCR) and WB analyses (Fig. 5B). Moreover, as shown in Fig. 5B, TGFβ1 mRNA and protein were almost undetectable in i-sEVs. TGFβ1, mRNA and protein were also found when a density gradient (OptiPrep) (27) was used to isolate e-sEVs (Supplementary Fig. S2). By cross-matching e-sEV transcripts and proteins significant positive correlations with the activation of matrix metalloproteinases and extracellular matrix organization (p-value = 9.17 × 10^−6^), the cytokine and chemokine signaling pathways (p-value = 4.7 × 10^−6^), IGF and platelet pathways (p-value = 5.03 × 10^−5^), the VEGF signaling pathway (p-value = 3.49 × 10^−4^) were detected (Fig. 5C).

### e-sEV treatment activates a specific gene signature

**Figure 6:**
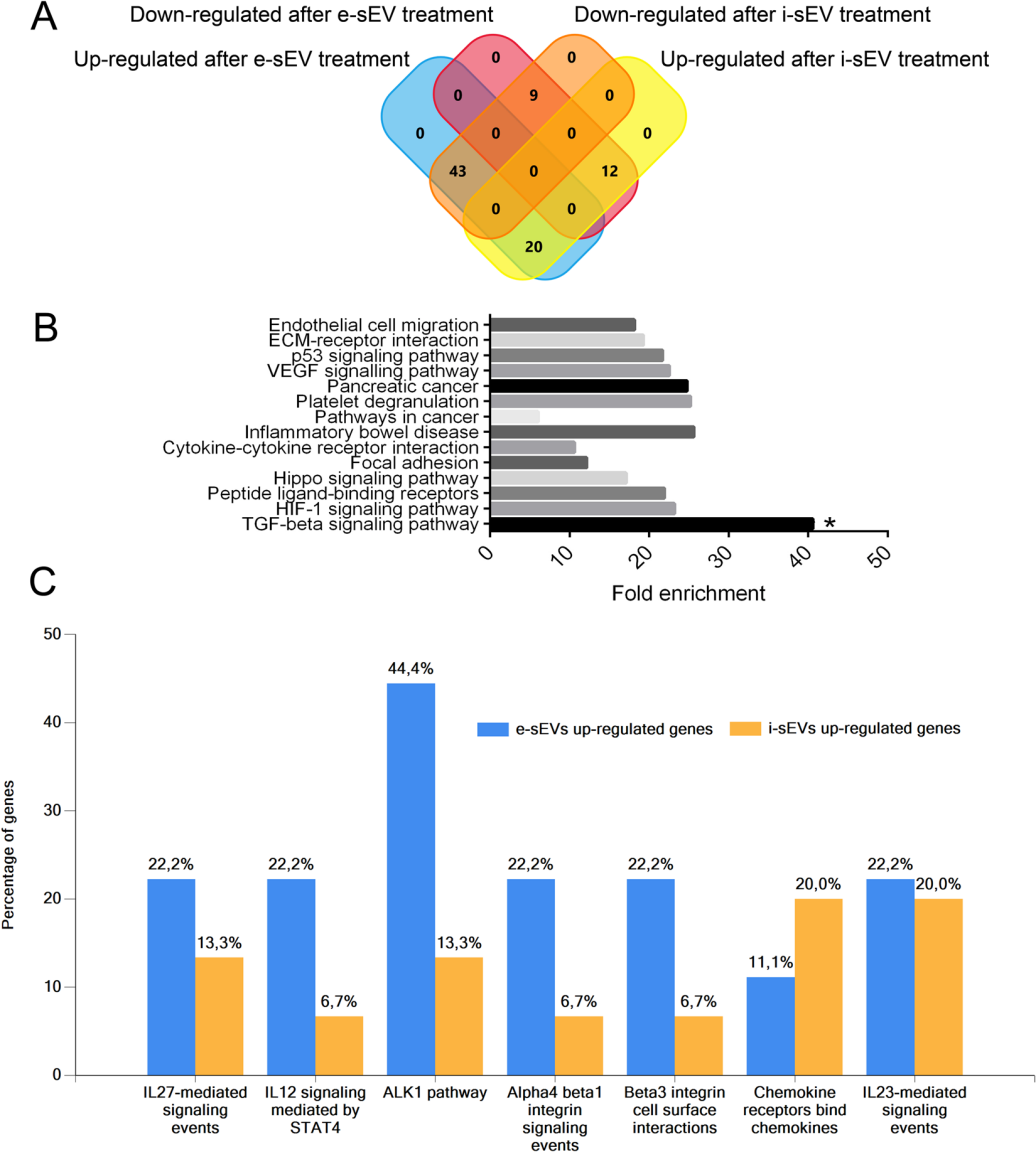
Gene pattern and representative biological pathways associated with e-sEV and i-sEV administration. (**A**) Representative Venn diagram of up-regulated and down-regulated mRNA in ECs treated with e-sEVs and i-sEVs. 43 genes up-regulated after e-sEV treatment were down-regulated upon i-sEV treatment; 12 genes down-regulated after e-sEV treatment were up-regulated after i-sEV treatment; 20 genes up-regulated upon e-sEV treatment were up-regulated upon i-sEV treatment and 9 genes down-regulated after e-sEV treatment were down-regulated upon i-sEV treatment. The list of genes is reported in Supplementary Table S4. (**B**) GO analysis on significantly over-expressed genes induced by e-sEV treatment (genes with a fold increase ≥3 were included). The significant enrichment of TGFβ1-associated pathway was identified. (**C**) The histogram represents the comparison of GO signaling pathways related to genes reported in the Venn diagram and Supplementary Tables S3 and S4. Columns represent the % of gene clusters involved in each signaling pathway.

In order to explore the mechanisms that account for e-sEV biological activity, an mRNA-angiogenesis microarray analysis was performed on ECs recovered from capillary-like structures formed in response to e-sEVs and i-sEVs. VEGF was used as a positive control. A discrete pattern of modulated genes emerged when e-sEV and i-sEV treatments were compared (Supplementary Tables S3 and S4 for each treatment and Table S5 for comparison between treatments). In particular, we found that 43 genes up-regulated (out of 84 analysed genes) upon e-sEV treatment were down-regulated upon i-sEV treatment (see Supplementary Table S5 and Fig. 6A). These data identify a specific gene signature, associated with e-sEVs, which supports divergence from i-sEV biological effects (Fig. 6A). We also compared the pattern of overexpressed genes activated in response to e-sEVs and VEGF (Supplementary Table S6) and found that, although the pro-angiogenic effects exerted by e-sEV and VEGF treatments were almost comparable, a different pattern of gene expression was noted. A Venn diagram, reported in Supplementary Fig. 3A, indicates the genes commonly modulated by these treatments. Gene Ontology (GO) was carried out, using DAVID GO-based functional analysis, on overexpressed transcripts (only transcripts with a fold increase values ≥3 were considered, see Supplementary Table S3) to dissect the molecular events associated with e-sEV treatment. As shown in Fig. 6B, an enrichment of genes associated with the TGFβ1 signaling pathway was found upon e-sEV treatment. As a matter of fact, the most representative clusters of genes activated in response to e-sEVs, but not i-sEVs, consist of genes involved in the ALK1 signaling pathway (Fig. 6C). These latter results further substantiate the transcriptomic analysis (Supplementary Table S3) which identified the *inhibitor of DNA binding I* (ID1) as one of the most highly expressed genes. Finally, GO functional analysis identified a diverse cluster of genes and related biological pathways (Supplementary Fig. S3) which is consistent with differences in the gene expression profile associated with VEGF and e-sEV treatment.

### e-sEV treatment leads to the formation of a SMAD1–5 transcriptional complex which binds the ID1 promoter

**Figure 7:**
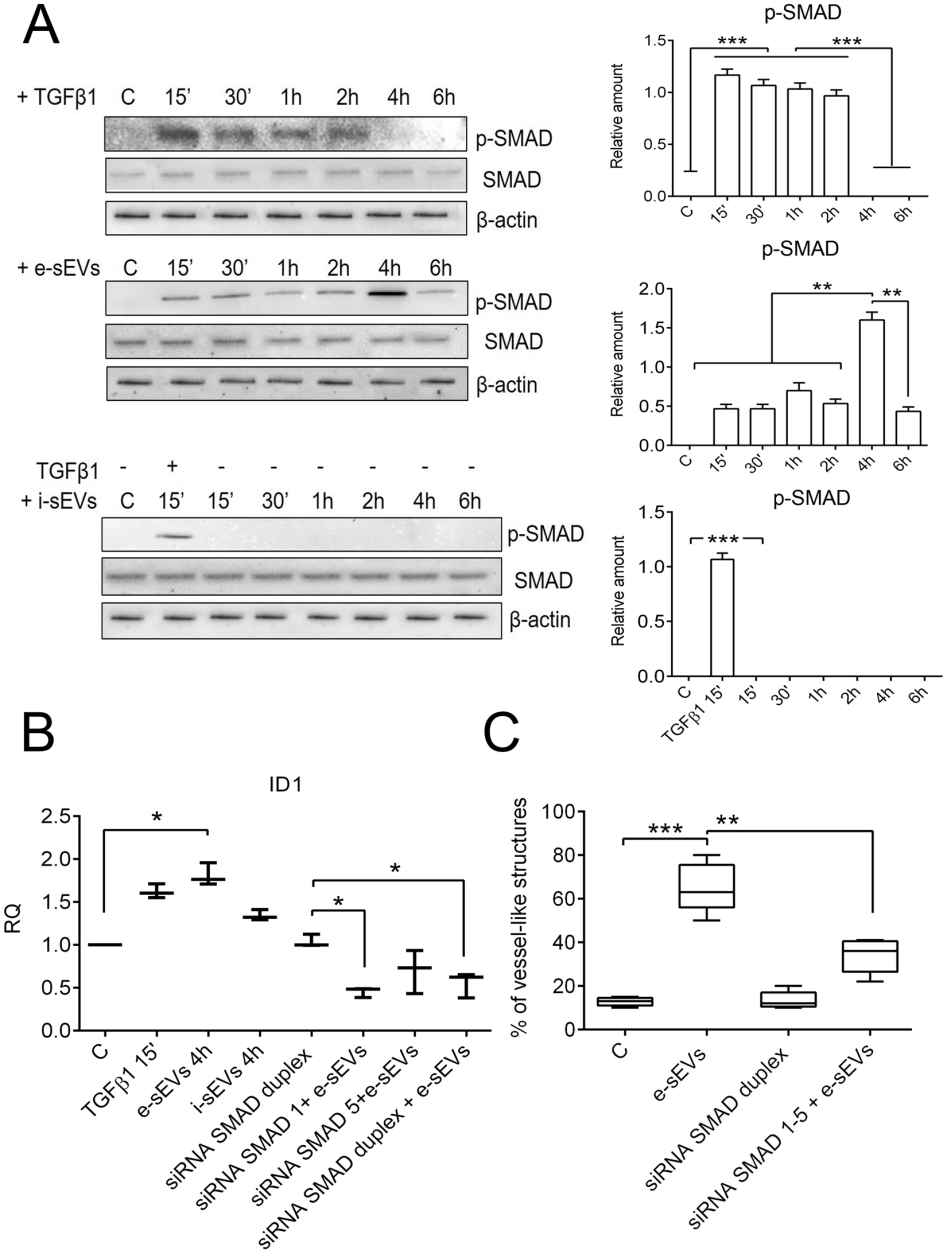
e-sEVs, but not i-sEVs, induce SMAD1-5 activation and lead to the formation of a SMAD1/5/ID1 transcriptional complex. (**A**) Representative SMAD1-5 phosphorylation. ECs treated for the indicated time with TGFβ1, e-sEVs and i-sEVs were lysated and analyzed by WB for p-SMAD 1/5, SMAD 1/5 and normalized to β-actin content. The results are representative of four different experiments (*n* = 4) **p < 0.01; ***p < 0.001 (One-way ANOVA followed by Tukey’s multicomparison test). (**B**) ChIP analysis of SMAD 1/5 binding to the ID1 promoter region using ECs treated for four hours with e-sEVs and i-sEVs and 15 minutes with TGFβ1. RQ values of RT-PCR are reported. Selected siRNAs for SMAD1-5 (see Supplementary Fig. S4) were transfected for 48 hours and ChIP analysis was performed as described above. ECs transfected with duplex siRNAs, alone or in combination with e-sEVs, were used as controls. (n = 4) *p < 0.05 C vs TGFβ1 and e-sEVs treatment (One-way ANOVA followed by Tukey’s multicomparison test). (**C**) *In vitro* quantitative analysis of the % of vessel-like structures formed (tubulogenesis assay) upon siRNA SMAD duplex transfection alone or in combination with e-sEVs. (n = 4). **P < 0.01 e-sEVs vs siRNA SMAD1-5 + e-sEVs; ***p < 0.001, C vs e-sEVs, (One-way ANOVA followed by Tukey’s multicomparison test).

It is widely recognized that the engagement of small mother against decapentaplegic (SMAD)1–5 by ALK1 is a crucial step for TGFβ1-mediated angiogenesis^32^. The activation of SMAD1–5 was therefore analyzed in ECs treated with sEVs and compared to TGFβ1 to further confirm the GO analysis. As shown in Fig. 7A, rapid and transient SMAD1–5 phosphorylation occurred upon TGFβ1 treatment. By contrast, e-sEV treatment led to delayed SMAD1–5 phosphorylation picking at 4 hours. It is worth noting that no SMAD1–5 phosphorylation was detected when i-sEVs were used. We thus sought to evaluate SMAD1–5 transcriptional activity on the ID1 promoter. A chromatin immunoprecipitation (ChIP) analysis was performed, for this purpose, using both e-sEVs and i-sEVs. As shown in Fig. 7B, only e-sEVs were able to induce the formation of a SMAD1–5 transcriptional complex which binds to the ID1 promoter, which is consistent with SMAD1–5 phosphorylation. siRNA technology was also used to confirm these data (Supplementary Fig. S4). As shown in Fig. 7B, SMAD1-5 knock-down abolished e-sEV-mediated SMAD1-5 transcriptional complex formation as well as binding to the ID1 promoter. This resulted in the inhibition of e-sEV-mediated tube-like structure formation (Fig. 7C).

## Discussion

EVs, that were first described decades ago^33^, have more recently attracted particular attention because their paracrine and endocrine action have been recognized^34^. Stem cell-derived EVs are the most studied EVs^20, 35^ as they possess the vast potential to impact upon both tissue engineering and regenerative medicine. EV biological activity has been linked to the transfer of bioactive lipids, proteins, receptors, mRNAs and miRNAs that may change the phenotype and function of recipient cells^36–38^. However, the past two decades have seen EVs also being detected in body fluids^23, 24^ serum which are easily accessible sources of EVs that are derived from different cell types^21, 39^. Data obtained by clinical studies revealed that about 80% of circulating EVs derive from platelets^40^. Although all cell types are able to release EVs^13, 41^, it has been reported that only a small subpopulation of circulating EVs are of endothelial origin^42^. We herein demonstrate that serum from healthy donors contains EVs which mainly express endothelial and platelets markers and, more importantly, display pro-angiogenic potential both *in vitro* and *in vivo*. *In vivo*, this is particularly evident from the analysis of capillary density which demonstrated that e-sEVs led to a doubling of the capillary density as compared to normal, while data from the Laser Doppler perfusion are less evident because this technique does not allow detection of profound vascular districts. Based on a functional *in vitro* “potency assay”, we identified a set of biologically active and inactive sEVs and were able to predict their *in vivo* angiogenic potential. Moreover, it has been shown herein that e-sEVs improved large vessel reperfusion and protected muscle from ischemia-induced damage in a preclinical model of acute hind limb ischemia. This data add further to the role of serum-derived exosomes described in the ischemia-reperfusion heart model^25^.

It is known that a number of soluble factors are released locally and actively participate in muscle regeneration after ischemia *in vivo* ^43, 44^. In particular, IGF1 has been shown to contribute to this process *in vivo* ^45^. In this work, proteomic analysis has demonstrated that e-sEVs are enriched in IGF1 and network analyses have revealed a significant positive correlation with the IGF pathway. No necrotic areas were found in the ischemic muscles of e-sEV-treated mice. This suggests that e-sEV treatment, rather than inducing regenerating signals to damaged muscles, is able to rapidly improve vascular remodeling and protect muscles against ischemia-induced tissue injury. Indeed, it has been extensively reported that one of the important components for tissue repair is the formation of new blood vessels^46^. Several studies have described the contribution of EVs, released from different stem cells types, in promoting angiogenesis^18, 37^, while miRNAs, mRNA and protein carried by EVs have been implicated in this process^20, 47^. Besides stem cells, platelet-derived EVs have also been reported to promote proliferation, survival, migration and the formation of capillary-like structures *in vitro* ^48^ and angiogenesis in ischemic rat hearts *in vivo* ^49^. However, answers to how EVs play a part in intercellular communication and how sEVs concomitantly contribute to target cell biological response are still not forthcoming and remain major challenges for the future. We herein demonstrate that e-sEVs are enriched in angiogenic modulators, in terms of both proteins and mRNAs. Moreover, database analysis demonstrates that TGFβ1 was expressed as mRNA and protein in e-sEVs, suggesting that TGFβ1 signaling may be relevant in inducing a fast angiogenic switch, resulting in muscle protection against ischemia-reperfusion damage in our preclinical model.

Several data indicate that the biological response of ECs to TGFβ1 might be either stimulatory or inhibitory, depending on the cell status and on microenvironment-mediated stimuli^50^. TGFβ1 signaling is initiated by binding to the constitutively active type II serine-threonine kinase receptor which, in turn, recruits and phosphorylates the type I receptor, the so called activin receptor-like kinese 5, ALK5^32^. ALK5 is the most widely expressed receptor. However, at sites of active angiogenesis^51^, ECs highly express a different type I serine-threonine kinase receptor, denoted ALK1^50^. The co-expression of both ALK1 and ALK5 led to the enrollment of varying SMAD family members; SMAD1–5 for ALK1 and SMAD2–3 for ALK5^32^. As the result of such differential activation, discrete target gene regulation takes place and leads to a variety of biological effects^52^. In fact, ALK5 inhibits EC migration *in vitro* ^53^ and angiogenesis *in vivo* ^54^ by inducing the expression of fibronectin and the plasminogen activator inhibitory type I (PAI-1), while ALK1 induces cell cycle progression by controlling the expression of ID1^55, 56^. It is worth noting that a comparison of GO functional analyses between e-sEV and i-sEV-treated cells revealed a specific gene signature, which relies on the TGFβ1-mediated pathway, that supports the role of TGFβ1 in the e-sEV mechanism of action. As a proof of concept, e-sEV treatment, but not i-sEV, led to SMAD1–5 phosphorylation and the formation of a SMAD1-5 transcriptional complex which bound to the ID1 promoter, while SMAD1-5 knock down prevented ID1 gene transcription and e-sEV-mediated tube-like structure formation. Further to these observations, GO functional analyses revealed an enrichment in the genes involved in the ALK1 pathway and linked to the activation phase of angiogenesis, but only in cells treated with e-sEVs.

The results provided herein therefore support the possibility that the e-sEV-mediated activation of the TGFβ1/ALK1/ID1 cascade may provide a fine tuning angiogenic response which balances the vascular remodeling process and protects muscles against ischemia-induced damage. This would be particularly relevant if e-sEVs could be exploited in a clinical setting, such as CLI. Indeed, CLI is considered the leading cause of non-traumatic amputations in the western world and brings with it a significant health burden globally. Surgical and endovascular treatments are currently considered to be the front-line in avoiding limb loss in humans^57^, however the recurrence of symptoms is a common problem^58^. Alternative treatment options are still limited also for patients to whom surgery has failed to improve long-term outcomes^59^. Clinical trials, including VEGF-A administration for therapeutic angiogenesis, have not been successful^60^. The establishment of a fine balance between VEGF and additional pro-angiogenic/stabilization pathways has recently been suggested as a means to improve the formation of a functional vascular network^61^. Interestingly, a different pattern of gene expression was found when GO functional analyses in VEGF and e-sEV treated cells were compared. One may speculate that e-sEVs and VEGF could become an alternative combined treatment with which to improve the formation of a functional vascular network. However, the positive correlation with the VEGF signaling pathway that was detected by cross-matching e-sEV transcripts and proteins means that e-sEVs may theoretically already be able to exert such a combinatory effect by themselves.

Overall, we have shown that EVs displaying pro-angiogenic potential can be obtained from the serum of healthy donors. We have identified a simple *in vitro* test to predict serum-EV angiogenic potential *in vivo*. The GO-based functional analysis of ECs recovered from the capillary-like structures formed in response to i-sEVs and e-sEVs have identified a gene signature which sheds light on their varying biological activity. Finally, we have identified the TGFβ1/ALK1/ID1 cascade as the most relevant mechanism for e-sEV biological activity, which is consistent with data obtained from the GO functional analysis. More importantly, our data provide the scientific rationale for the use of sEVs as an alternative therapeutic option for vascular remodeling, better local perfusion and protection against muscle damage in patients with acute hind limb ischemia.

## Methods

### Study approval

Animal studies were conducted in accordance with the Italian National Institute of Health Guide for the Care and Use of Laboratory Animals. All procedures were approved by the Ethics Committee of the University of Turin and the Italian Health Ministry (authorization number:490/2105-PR). Mice were housed according with the Federation of European Laboratory Animal Science Association Guidelines. All experiments were performed in accordance with relevant guidelines and regulations.

### Vesicle isolation and characterization

Human serum from healthy blood donors (n = 18) was provided by the Blood Bank of “Città della Salute e della Scienza di Torino”, after informed consent and approval by the internal Review Board of the Blood Bank. Informed consent was obtained by the Blood Bank of “Città della Salute e della Scienza di Torino” from all participants. sEVs from each donor were obtained from 110 ml serum bags. Details are reported in (Supplementary Information).

### Floating density gradient separation

Floating density separation on iodixanol (Optiprep from Sigma) was modified, from Kowal *et al*.^28^, in order to obtain larger centrifugation volumes with sufficient amounts of e-sEVs for functional studies. Details are reported in (Supplementary Information). 35 ng of proteins were loaded for the WB analysis. Supersignal West Femto Maximum Sensitivity Substrate (Thermo Scientific, Rockford, IL, USA) was used for detection. 3 ng of total RNA was used for the RT-PCR analysis. Details are reported in (Supplementary Information).

### Guava FACS analysis and Nanoparticle tracking analysis

sEV FACS analysis was performed using a Guava easyCyte™ Flow Cytometer (Millipore, Germany) as previously described^5, 62^. Specific cell markers were used. sEVs were also analyzed using the Nanosight LM10 system (Nanosight Ltd., Amesbury, UK) as previously described^62^. Details are reported in (Supplementary Information).

### sEV angiogenic assay

In preliminary studies, a dose response curve was performed to evaluate the number of sEVs needed to obtain the best biological response in human microvascular endothelial cells (HMEC)^5^. The acronym ECs will be used throughout the study. It was found, using 4 different sEV samples, that 5 × 10^4^ sEVs/target cells was the most effective sEV dose. 5 × 10^4^ sEVs/target cells were therefore used throughout the *in vitro* study^63^. sEVs from single samples were thus evaluated for their pro-angiogenic activity using BrdU^64^ and *in vitro* tubulogenesis assays^37^. Negative and positive controls were used to evaluate sEVs angiogenic potency (for *BrdU assay*: negative control was medium w/o FCS; positive control was with 10% FCS; *in vitro angiogenesis assay*: positive control was 10 ng/ml of VEGF). The following formula was applied:

$$ \% \,effect=(\frac{sample\,value-neg\,ctrl\,value(0 \% )}{pos\,ctrl\,value(100 \% )-neg\,ctrl\,value\,})\times 100.$$

Values exceeding 50% of VEGF pro-angiogenic capability (for both assays) were considered as making sEVs efficient.

### *In vivo* angiogenesis assay

Angiogenesis was assessed as previously described^5, 37^. Briefly ECs (1 × 10^6^ cells/injection) were incubated overnight with sEVs (5 × 10^10^ EVs per 1 × 10^6^ of ECs) and injected subcutaneously in severe combined immunodeficiency (SCID) mice. The vessel lumen area was determined as previously described^5^. Details are reported in (Supplementary Information).

### Murine model of acute hind limb ischemia and blood flow monitoring

C57 mice (Charles River Laboratories), age 7 to 8 weeks were used. Hind limb ischemia was obtained as described^19, 31, 65, 66^. In preliminary experiments, different sEV numbers and e-sEV administration routes were evaluated (not shown). The latter included intravenous (iv) and intramuscular (im) alone or differently combined. We found that the best administration route and e-sEV number, in terms of animal distress and response to treatment, was 2 × 10^10^ sEVs: 1 × 10^10^ administrated immediately after intervention (T0) iv, 0.5 × 10^10^ im on day one (T1) and day two (T2). Animals were sacrificed on day 7 (T7) for histological analysis. Details are reported in (Online Data Supplement). Hind limb blood flow was measured using a Laser Doppler Blood Flow (LDBF) analyzer (PeriScan PIM 3 System, Perimed, Stockholm, Sweden), before and after surgery and at days 3 and 7 after surgery as previously described^65^. Details are reported in (Supplementary Information).

### *In vivo* assessment of limb function

Semiquantitative estimation (repeated measures were analyzed with ANOVA and Newman-Keuls Multiple Comparison test) of foot damage was performed serially using the following classification: 3 = dragging of foot (foot necrosis), 2 = no dragging but no plantar flexion (foot damage), 1 = plantar flexion but no toe flexion (toe damage), and 0 = flexing the toes to resist gentle traction on the tail (no damage)^67^.

### Evaluation of capillary density and inflammatory cells

Capillary density and inflammatory cells were quantified within gastrocnemius muscles using immunofluorescence analysis. Muscle samples were embedded in OCT compound (Bio-Optica) and processed as previously described^66^. Cryosections of the ischemic limbs were stained with rat anti-mouse CD14 primary antibody (PharMingen), while anti-rat Alexa Fluor Texas Red (Molecular Probe) was used as secondary antibody. Details are reported in (Supplementary Information).

### Histology

The gastrocnemius muscle, from ischemic and non-ischemic limbs, was removed at day 7 after surgery. Tissue slices were stained with hematoxylin and eosin^65^. Details are reported in (Supplementary Information).

### Protein array

Proteins from different sEV preparations were extracted using NP40 lysis buffer (150 mM NaCl, 50 mM TRIS-HCl pH 8, 1% NP40) and quantified on a BCA protein assay (Thermo Fisher). The angiogenic protein profile was performed using Quantibody® Human Angiogenesis array 1000 (Ray Biotech). 25 µg of protein were loaded for each sample. Data were expressed as concentration (pg/ml ± SD). Functional annotation enrichment analysis was performed using Funrich V3 software and the geneMANIA plug in on Cytoscape. Details are reported in (Supplementary Information).

### Western Blot analysis

Proteins from different sEVs and from ECs, treated as indicated were extracted using the RIPA buffer (Sigma), then quantified. Thirty µg, for cells, and 10 µg, for EVs, were subjected to WB analysis as previously described^66^. Anti-p^ser^463-465-SMAD1-5 (Cell Signalling), SMAD1/5/9 (abcam), β-actin and TGFβ1 (St. Cruz Biotechnology Inc., Santa Cruz, CA) were used. Details are reported in (Supplementary Information).

### mRNA-angiogenic microarray profile

Total RNA from 4 e-sEV and 4 i-sEV samples was isolated using the All in One (Norgen, Thorold, ON, Canada) extraction method. cDNA was synthesized using the RT^2^ First Strand kit (SABiosciences) according to manufacturer’s instructions. Gene expression profiling, using the Angiogenesis RT^2^ Profiler PCR Array (PAHS 024, SA Biosciences), was performed on by loading 200 ng of cDNA for each sEV sample. The expression profile of 84 key genes in angiogenesis was analyzed (list of genes available on website: http://www.sabiosciences.com). Details are reported in (Supplementary Information). The angiogenesis RT^2^ Profiler PCR Array was also performed on tubule-like structures formed upon sEV administration (n = 6 controls; n = 6 VEGF; n = 4 e-sEVs; n = 4 i-sEVs). Changes in the gene expression of treated, with respect to untreated, ECs were reported as a fold increase/decrease ± SD. For e-sEV and i-sEV treatment, up-regulated transcripts with a fold increase ≥3 with respect to untreated ECs were used for further investigation. Statistical analysis was performed through One-way ANOVA, followed by Tukey’s post hoc or multiple comparison test. Data were further analyzed using Expression Suite and Funrich V3 Software. Functional annotation enrichment analysis was performed using Funrich V3 software and DAVID GO. Details are reported in (Supplementary Information).

### SMAD 1-5 siRNA transfection on ECs

siRNA transfection on ECs was performed using Hiperfect Transfection Reagent (Qiagen) according to specific manufacturer’s instructions for ECs. A gene-specific package of 4 pre-selected siRNAs for SMAD1 and SMAD5 (Qiagen) were used. Each siRNA targeted a specific sequence for SMAD1 (Supplementary Information). The knock-down of SMAD1 and SMAD5 was ascertained by comparing RQ values of ECs transfected with duplex siRNAs (negative control) or with siRNA for SMAD1–5 by RT-PCR. Silencing was carried out for 48 or 72 hours post-transfection. Details are reported in (Supplementary Information).

### Chromatin Immunoprecipitation (ChIP) Assay

A ChIP assay was performed on ECs treated with sEVs using Magna ChIP A kit (Millipore), according to the vendor’s instructions^63^. ECs, treated as indicated, were cross-linked with 1% formaldehyde and quenched before harvest and sonication. The sheared chromatin was immunoprecipitated with either the anti-p^ser^463-465-SMAD1-5 antibody or control IgG on protein G Sepharose magnetic beads. The eluted IP were processed as previously described^63^. Details are reported in (Supplementary Information). ChIP analysis was also performed in ECs transfected with siRNAs.

### Statistics

Results are expressed as mean ± SD or ± SEM, unless otherwise reported. Statistical analysis was carried out using One-way ANOVA, followed by Tukey’s post hoc or multiple comparison, Student t tests for 2-group comparison and Newman-Keuls Multiple Comparison Test where appropriate. The cut-off for statistical significance^68, 69^ was set at p < 0.05 (*p < 0.05, **p < 0.01, ***p < 0.001).

## Electronic supplementary material

Supplementary Information
