# Supplementary material for: Serum-derived extracellular vesicles (EVs) impact on vascular remodeling and prevent muscle damage in acute hind limb ischemia
Source: Sci Rep. 2017 Aug 15;7:8180. doi: 10.1038/s41598-017-08250-0 (PMC5557987; doi:10.1038/s41598-017-08250-0)
Supplement: Supplementary file 1 — Supplementary Information [file 41598_2017_8250_MOESM1_ESM.pdf]

Serum-derived extracellular vesicles (EVs) impact on vascular remodeling and prevent muscle damage in acute hind limb ischemia

Claudia Cavallari, Andrea Ranghino, Marta Tapparo, Massimo Cedrino, Federico Figliolini, Cristina Grange, Valentina Giannachi, Paolo Garneri, Maria Chiara Deregibus, Federica Collino, Pietro Rispoli, Giovanni Camussi, Maria Felice Brizzi.

SUPPLEMENTARY INFORMATION

DETAILED METHODS

Vesicle isolation and characterization

Human serum from healthy blood donors (n=18) was provided by the Blood Bank of "Città della Salute e della Scienza di Torino", after informed consent and approval by the internal Review Board of the Blood Bank. Informed consent was obtained by the Blood Bank of "Città della Salute e della Scienza di Torino" from all participants. sEVs from each donor were obtained from 110 ml serum bags by centrifuging serum at 3,000 g for 20 minutes to remove debris. The supernatant was subsequently ultracentrifuged at 100,000 for 2 hours at 4°C. EVs pellets were then re-suspended in a final volume of 1 ml of RPMI with 1% of DMSO and stored at -80°C. sEV were then thawed and used for biological assays and molecular analysis.

Floating density gradient separation

Floating density separation on iodixanol (Optiprep from Sigma) was modified, from Kowal et al.,[1] in order to obtain larger centrifugation volumes with sufficient amounts of e-sEVs for functional studies. The e-sEV pellets, obtained by differential ultracentrifugation, were directly re-suspended in 500 microliters of 60% iodixanol with 0.25M sucrose. Next, 1 ml of 30%, 15% and 5% iodixanol working solution (obtained from 60% iodixanol 0.25M sucrose by adding 10mM Tris, 1mM EDTA with a final pH of 7.4) were sequentially layered on top of the e-sEV/60% iodixanol suspension. The final volume of 10 ml was obtained by adding saline solution on top of the 5% iodixanol fraction. Tubes (10.4 ml polycarbonate bottles, Cat. N. 355603 Beckman Instruments, Palo Alto, CA) were submitted to ultracentrifugation at 350,000g for 1 hour at 4°C without a break in a Optima L-100K ultracentrifuge (Beckman Coulter) equipped with a type 90Ti rotor. The saline and 5% iodixanol fraction, which did not contain e-sEVs, were discarded and the 15, 30 and 60% fractions were recovered, diluted with PBS and submitted to ultracentrifugation at 100,000g for 1 hour at 4°C. The pellet of each fraction was then re-suspended in PBS with 1% DMSO for subsequent study. Floating vesicles were detected, using NanoSight, principally in the 30% fraction (70%) and, to a lesser minor extent, in the 15% fraction (30%). 35 ng of proteins were loaded for the WB analysis. Supersignal West Femto Maximum Sensitivity Substrate (product # 34096) (Thermo Scientific, Rockford, IL, USA) was used for detection. 3 ng of total RNA was used for the RT-PCR analysis.

Guava FACS analysis

sEV FACS analysis was performed using a Guava easyCyte™ Flow Cytometer (Millipore, Germany) as previously described[2,3]. sEVs from healthy donors were incubated with antibodies directed to specific markers for monocyte/macrophages (CD14, CD15), leukocytes (CD45), adhesion molecules (alpha6 integrin, CD44, CD29), endothelial cells (CD31, KDR) and platelets (CD42b, CD62P, P SEL). FITC or PE mouse non-immune isotypic IgG (Beckton Dickinson, Franklin Lakes, NJ) were used as controls. sEVs were incubated with specific or control isotype antibodies for 1 hour at 4°C and analyzed by FACS.

Nanoparticle tracking analysis

sEVs were analyzed using the Nanosight LM10 system (Nanosight Ltd., Amesbury, UK). Briefly, sEVs preparations were diluted (1:1000) in sterile 0.9% saline solution and analyzed by NanoSight LM10 equipped with the Nanoparticle Analysis System & NTA 1.4 Analytical Software. The number of total EVs for each patient was obtained by multiplying the value given by the instrument (microparticles/ml) by the dilution for the analysis and by the number of microliters in which sEVs were re-suspended[3].

sEV angiogenic assay

In preliminary studies, a dose response curve was performed to evaluate the number of sEVs needed to obtain the best biological response in human microvascular endothelial cells (HMEC)[2]. The acronym ECs will be used throughout the study. It was found, using 4 different sEV samples, that $5x10^4$ sEVs/target cells was the most effective sEV dose. $5x10^4$ sEVs/target cells were therefore used throughout the *in vitro* study[4]. sEVs from single samples were thus evaluated for their pro-angiogenic activity using BrdU[5] and *in vitro* tubulogenesis assays[6]. Negative and positive controls were used to evaluate sEVs angiogenic potency (for *BrdU assay*: negative control was medium w/o FCS; positive control was with 10% FCS; *in vitro angiogenesis assay*: positive control was 10 ng/ml of VEGF). The following formula was applied:
% effect $= \left(\frac{sample\ value - neg\ ctrl\ value\ (0\%)}{pos\ ctrl\ value\ (100\%) - neg\ ctrl\ value}\right) \times 100$. Values exceeding 50% of VEGF proangiogenic capability (for both assays) were considered as making sEVs efficient.

In vivo angiogenesis assay

Angiogenesis was assessed by measuring the growth of blood vessels from subcutaneous tissue into a solid gel of basement membrane, as previously described[2,7]. First, ECs (1×10^6 cells/injection) were incubated overnight with sEVs (5×10^{10} EVs per 1×10^6 of ECs). Male severe combined immunodeficiency (SCID) mice (6 wks old) were then injected subcutaneously with 0.5 ml of ice-cold BD Matrigel Matrix Growth Factor Reduced (BD Biosciences), which had been mixed with pre-stimulated ECs. An equal number of non-stimulated ECs was used as a negative control. The Matrigel plugs were excised 7 days later and fixed in 4% paraformaldehyde for 8 hours. Matrigel-containing paraffin sections (5–8 μm thick) were stained using the trichrome stain method. [2] The vessel lumen area was determined as previously described.[2]

Murine model of hind limb ischemia

C57 mice (Charles River Laboratories), age 7 to 8 weeks and weighing 18 to 22 g, were anesthetized via the intramuscular injection of zolazepam 80 mg/Kg. The animals were closely monitored postoperatively and analgesia with Ketorolac (5 mg/Kg) was administered if required. A small skin incision was made under sterile conditions overlying the middle portion of the left hind limb of each mouse. The proximal end of the left femoral artery and the distal portion of the saphenous artery were ligated, dissected and excised. The overlying skin was then closed using a sterilized 6-0 silk suture[8]. In preliminary experiments, different sEV numbers (ranging from $1x10^9$ to $1x10^{11}$) and e-sEV administration routes were evaluated (not shown). The latter included intravenous (iv) and intramuscular (im) alone or differently combined. We found that the best administration route and e-sEV number, in terms of animal distress and response to treatment,

was $2x10^{10}$ sEVs: $1x10^{10}$ administrated immediately after intervention (T0) iv, $0.5x10^{10}$ im on day one (T1) and day two (T2). Animals were sacrificed on day 7 (T7) for histological analysis.

Hind limb blood flow monitoring

After anesthesia, mice were placed on a heating plate at 37 °C for 5 min to minimize temperature variations. Hind limb blood flow was measured using a Laser Doppler Blood Flow (LDBF) analyzer (PeriScan PIM 3 System, Perimed, Stockholm, Sweden), immediately before and after surgery and at days 3 and 7 after surgery. LDBF analysis was performed on hind limbs and feet. Blood flow was reported as changes in the laser frequency using different color pixels. Images were analyzed to quantify blood flow using ROIs (regions of interest) drawn freehand. To avoid data variations that may be caused by ambient light and temperature, hind limb blood flow was expressed as the ratio of left (ischemic) to right (non-ischemic) LDBF[8].

Evaluation of capillary density and inflammatory cells

Capillary density and inflammatory cell recruitment was quantified within gastrocnemius muscles using immunofluorescence analysis. Muscle samples were embedded in OCT compound (Bio-Optica) and snap-frozen in liquid nitrogen. Tissue slices (5 µm in thickness) were prepared and capillary endothelial cells identified by immunofluorescence using a goat polyclonal antibody against mouse CD31 (Santa Cruz Biotechnology Inc., Santa Cruz, CA); anti-goat Alexa Fluor 488 (Molecular Probe) was used as secondary antibody. Hoechst was added for nuclear staining. Fifteen randomly chosen microscopic fields, from three different sections in each tissue block, were examined for the capillary endothelial cell count. Capillary density was expressed as the number of CD-31-positive features per high power field (HPF) ± SEM (Magnification: x400). Cryosections of the ischemic limbs were stained with rat anti mouse CD14 primary antibody (PharMingen), while anti-rat Alexa Fluor Texas Red (Molecular Probe) was used as secondary antibody. Dapi was added for nuclear staining. Immunofluorescence was performed on six ischemic non-treated hind limbs and on six e-sEV-treated ischemic hind limbs. CD14 positive cells were counted in ten randomly chosen microscopic fields for each sample (magnification: x400) and expressed as mean per high power field (HPF) ± SEM.

Histology

The gastrocnemius muscle, from ischemic and non-ischemic limbs, was removed at day 7 after surgery, immediately fixed with 4% paraformaldehyde (Sigma) for 8 hours and then embedded in paraffin. Tissue slices were stained with hematoxylin and eosin. Slides were examined under light microscopy at ×200 magnification. Images were acquired from all the injured areas of the ischemic limb sections for the total muscle fiber count. Random images from the total, non-ischemic limb section were considered as a control[9].

Protein array

Proteins from different sEV preparations were extracted using NP40 lysis buffer (150 mM NaCL, 50 mM TRIS-HCl pH 8, 1% NP40) and quantified on a BCA protein assay (Thermo Fisher). The angiogenic protein profile was performed using Quantibody® Human Angiogenesis array 1000 (Ray Biotech) which contains a combination of two non-overlapping arrays to quantitatively measure the expression of 60 human angiogenic proteins. 25 µg of protein were loaded for each sample. Data were subjected to background subtraction and protein concentration was obtained from the different standard curve of the array. Data were expressed as concentration (pg/ml) ±SD. Functional annotation enrichment analysis was performed using Funrich V3 software and the geneMANIA plug in on Cytoscape**.**

Western Blot analysis

Proteins from different s-EVs and from ECs, treated with either TGFβ1, e-sEV or i-sEV, were extracted using the RIPA buffer (Sigma), supplemented with proteases and phosphatase inhibitors (Sigma) and then quantified using a Bradford assay (Biorad). Thirty µg, for cells, and 10 µg, for

EVs, were subjected to SDS-PAGE, transferred into nitrocellulose membranes and underwent immunoblotting with antibodies against anti-p^{ser}463-465-SMAD1-5 (Cell Signalling), SMAD1/5/9 (abcam), β-actin and TGFβ1 (St. Cruz Biotechnology Inc., Santa Cruz, CA). Densitometric analyses, performed on Image Lab software (Biorad), were used to calculate the differences in the fold induction of protein levels, which were normalized to SMAD1/5/9 and β-actin[10]. Values are reported as relative amounts.

mRNA-angiogenic microarray profile

Total RNA from 4 e-sEV and 4 i-sEV samples was isolated using the All in One (Norgen, Thorold, ON, Canada) extraction method. RNA concentration was measured using the NanoDrop1000 spectrophotometer. cDNA was synthesized using the RT^2 First Strand kit (SABiosciences) according to manufacturer’s instructions. Gene expression profiling, using the Angiogenesis RT^2 Profiler PCR Array (PAHS 024, SA Biosciences), was performed by loading 200 ng of cDNA for each sEV sample. The expression profile of 84 key genes in angiogenesis was analyzed (list of genes available on website: http://www.sabiosciences.com). Quantitative RT- PCR (qRT-PCR) was conducted using the StepOne Plus^{TM} System (AB Applied Biosciences). Relative gene expression was determined using the $\Delta\Delta C_T$ method. The angiogenesis RT^2 Profiler PCR Array was also performed on tubule-like structures formed upon sEV administration. ECs were harvested and purified using the RNeasy Mini kit (Qiagen, Germany), and processed using the Angiogenesis RT^{2}Profiler PCR Array. Five endogenous control genes; beta-2-microglobulin (B2M), hypoxanthine phosphoribosyltransferase (HPRT1), 60S acidic ribosomal protein P0 (RPLP0), glyceraldehyde-3-phosphate dehydrogenase (GAPDH), and b-actin (ACTB), all present on the PCR array, were used for normalization. The fold-change for each treated sample corresponded to $2^{-\Delta\Delta Ct}$, relative to the control sample. Changes in the gene expression of treated, with respect to untreated, ECs were reported as a fold increase/decrease ± SD. For e-sEV and i-sEV treatment, up-regulated transcripts with a fold increase ≥ 3 with respect to control samples (untreated ECs) were used for further investigation. Data were further analyzed using Expression Suite and Funrich V3 Software. Functional annotation enrichment analysis was performed using Funrich V3 software and DAVID GO.

SMAD 1/5 siRNA transfection on ECs

siRNA transfection on ECs was performed using Hiperfect Transfection Reagent (Qiagen) according to specific manufacturer’s instructions for ECs. A gene-specific package of 4 pre-selected siRNAs for SMAD1 and SMAD5 (Qiagen) were used at 10 nM. Each siRNA targeted a specific sequence for SMAD1: siRNA1 (CTGGTGCTCTATTGTCTACTA), siRNA 2 (CTCCCAATAGCAGTTACCCAA); siRNA 3 (TTAGCTCAGTTCCGTAACTTA); siRNA 4 (AACCGGAATTCCACTATTGAA) and for SMAD5: siRNA 1 (AGCCTAAACATTGGTGTTCAA) siRNA 2 (AACATCTGATGTACCAATTTA) siRNA3 (CTGAGGTGAAATCCAGAGTAA); siRNA 4 (AGGCGTGTTATTGATCTGCTA). The knock-down of SMAD1 and SMAD5 was ascertained by comparing RQ values of ECs transfected with duplex siRNAs (negative control) or with siRNA for SMAD1-5 by real time PCR. Silencing was carried out for 48 or 72 hours post-transfection.

Chromatin Immunoprecipitation (ChIP) Assay

A ChIP assay was performed on ECs treated with sEVs, as indicated in the Results section, using Magna ChIP A kit (Millipore), according to the vendor’s instructions[4]. Briefly, ECs, treated as indicated, were cross-linked with 1% formaldehyde and quenched before harvest and sonication. The sheared chromatin was immunoprecipitated with either the anti-p^{ser}463-465-SMAD1-5 antibody or control IgG on protein G Sepharose magnetic beads. The eluted IP were digested with proteinase K, while DNA was extracted and underwent real time PCR with primers specific for ID1 promoter region: sense, 5’-CAGTTTGTCGTCTCCATG-3’; antisense, 5’-TCTGTGTCAGCGTCTGAA-3’ and GAPDH: sense, 5’-TGGAAGGACTCATGACCACAGT-3’; antisense, 5’-CATCACGCCACAGTTTCCC-3’ as the housekeeping gene control. RQ values were considered using untreated EC samples as internal control. ChIP analysis was also performed in ECs transfected with duplex siRNAs or with siRNA for SMAD1-5.

Statistics

Results are expressed as mean ± SD or ± SEM, unless otherwise reported. Statistical analysis was carried out using 1-way ANOVA, followed by Tukey's post hoc or multiple comparison, Student t tests for 2-group comparison and Newman-Keuls Multiple Comparison Test where appropriate. The cut-off for statistical significance was set at $p<0.05$ (*$p<0.05$, **$p<0.01$, ***$p<0.001$).

REFERENCES

1. Kowal J, Arras G, Colombo M, Jouve M, Morath JP, Primdal-Bengtson B, Dingli F, Loew D, Tkach M, Théry C. Proteomic comparison defines novel markers to characterize heterogeneous populations of extracellular vesicle subtypes. *Proc Natl Acad Sci U S A*. 2016;113:E968-77.

2. Lopatina T, Bruno S, Tetta C, Kalinina N, Porta M, Camussi G. Platelet-derived growth factor regulates the secretion of extracellular vesicles by adipose mesenchymal stem cells and enhances their angiogenic potential. *Cell Commun Signal*. 2014;12:26.

3. Figliolini F, Cantaluppi V, De Lena M, Beltramo S, Romagnoli R, Salizzoni M, Melzi R, Nano R, Piemonti L, Tetta C, Biancone L, Camussi G. Isolation, characterization and potential role in beta cell-endothelium cross-talk of extracellular vesicles released from human pancreatic islets. *PLoS One*. 2014;9.

4. Lombardo G, Dentelli P, Togliatto G, Rosso A, Gili M, Gallo S, Deregibus MC, Camussi G, Brizzi MF. Activated Stat5 trafficking Via Endothelial Cell-derived Extracellular Vesicles Controls IL-3 Pro-angiogenic Paracrine Action. *Sci Rep*. 2016;6:25689.

5. Bussolati B, Deambrosis I, Russo S, Deregibus MC, Camussi G. Altered angiogenesis and survival in human tumor-derived endothelial cells. *FASEB J*. 2003;17:1159–1161.

6. Deregibus MC, Cantaluppi V, Calogero R, Iacono M Lo, Tetta C, Bruno S, Bussolati B, Camussi G, Dc W, Biancone L. Endothelial progenitor cell derived microvesicles activate an angiogenic program in endothelial cells by a horizontal transfer of mRNA. 2012;110:2440–2448.

7. Couffinhal T, Silver M, Zheng LP, Kearney M, Witzenbichler B, Isner JM. Mouse model of angiogenesis. *Am J Pathol*. 1998;152:1667–1679.

8. Ranghino A, Cantaluppi V, Grange C, Vitillo L, Fop F, Biancone L, Deregibus MC, Tetta C, Segoloni GP CG. Endothelial progenitor cell-derived microvesicles improve neovascularization in a murine model of hindlimb ischemia. *Int J Immunopathol Pharmacol*. 2012;25:75–85.

9. Sudo M, Kano Y. Myofiber apoptosis occurs in the inflammation and regeneration phase following eccentric contractions in rats. *J Physiol Sci*. 2009;59:405–412.

10. Gallo S, Gili M, Lombardo G, Rossetti A, Rosso A, Dentelli P, Togliatto, G Deregibus MC, Taverna D, Camussi G, Brizzi MF. Stem cell-derived, microRNA-Carrying extracellular vesicles: A novel approach to interfering with mesangial cell collagen production in a hyperglycaemic setting. *PLoS One*. 2016;11:1–18.

Supplementary TABLE S1

	Serum EVs	
	**MARKERS**	**%**
Monocyte/ Leucocytes	**CD14**	**10.5±0.7**
	**CD15**	**7.8±0.9**
	**CD45**	**4.5±0.3**
Adhesion	**ALPHA6**	**11.3±1.1**
	**CD44**	**11.10±1.2**
	**CD29**	**16.5±1.1**
Endothelial	**CD31**	**35.1±2**
	**KDR**	**20.5±0.9**
Platelet	**CD62P**	**20.1±2.2**
	**P SEL**	**28.8±3.1**
	**CD42B**	**22±1.4**

Table S1. **Characterization of sEV surface antigens.** Different sEV surface markers have been analyzed by Guava FACS analysis. Table summarizes the percentage of sEV positivity for platelets, endothelial cells, monocytes/leukocytes and cell adhesion markers. Data represent the average percentage value of different samples ± SD (n=5).

Supplementary FIGURE S1

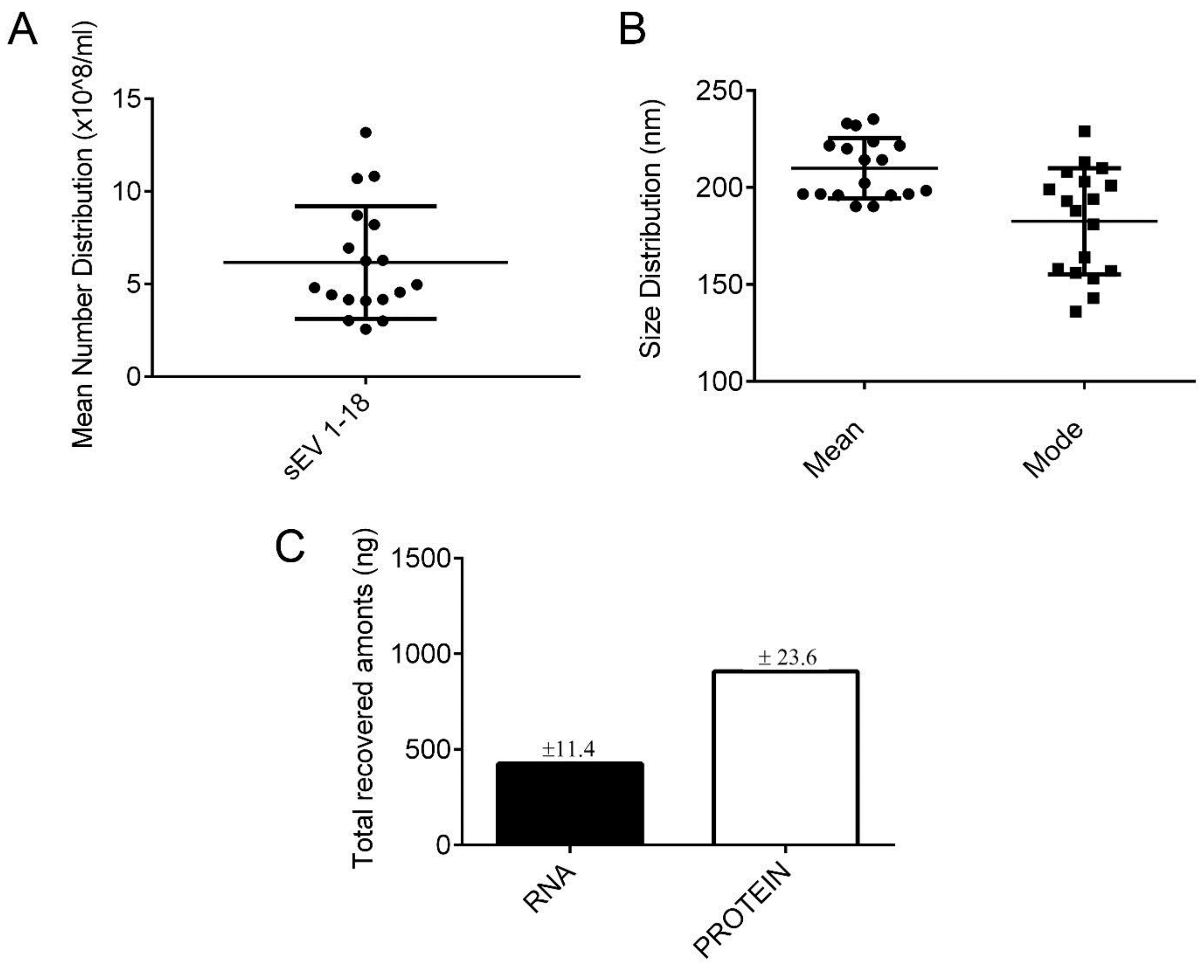

Figure S1. sEV profile. (A) Number distribution of sEV samples by NTA. Dots represent mean quantification of each measured sEV sample (n=18). (B) NTA size distribution with mean (210 nm) and mode (183 nm) size values for each sEV sample (n=18). (C) RNA and protein quantification of sEVs starting from $2x10^{11}$ total particles. Data represent the mean ±SD.

Supplementary TABLE S2

mRNA	*Average Ct*			*mRNA*	*Average Ct*		
**CXCL8**	25.36	±	0.63	**CXCL10**	28.46	±	1.05
**PF4**	25.71	±	0.66	**ANPEP**	28.48	±	0.83
**PROK2**	27.21	±	1.16	**THBS1**	28.49	±	0.81
**NRP1**	27.31	±	0.85	**IFNG**	28.49	±	1.16
**EFNB2**	27.35	±	0.59	**COL4A3**	28.49	±	1.19
**PLG**	27.38	±	0.93	**EFNA1**	28.52	±	0.61
**VEGFA**	27.61	±	1.33	**IGF1**	28.54	±	0.73
**CXCL9**	27.64	±	0.87	**IL1B**	28.54	±	0.48
**FLT1**	27.77	±	0.73	**VEGFB**	28.54	±	0.55
**CXCL1**	27.81	±	0.63	**COL18A1**	28.55	±	0.61
**TGFB1**	27.82	±	0.59	**ANGPT2**	28.58	±	0.78
**CXCL6**	27.82	±	0.90	**TNF**	28.60	±	0.73
**HIF1A**	27.84	±	0.96	**FN1**	28.60	±	1.30
**EDN1**	27.86	±	0.78	**ENG**	28.63	±	0.56
**NOTCH4**	27.89	±	1.03	**SERPINE1**	28.65	±	0.67
**TGFA**	27.90	±	0.62	**MMP2**	28.67	±	0.76
**TGFB2**	27.92	±	1.04	**PLAU**	28.67	±	0.51
**ITGB3**	27.95	±	0.69	**TIE1**	28.69	±	0.85
**IL6**	27.96	±	1.26	**LEP**	28.72	±	0.74
**ANGPT1**	27.99	±	1.06	**TIMP2**	28.73	±	0.77
**HPSE**	27.99	±	0.82	**AKT1**	28.73	±	0.45
**TEK**	28.00	±	0.77	**CDH5**	28.73	±	0.44
**PGF**	28.01	±	0.56	**SPHK1**	28.78	±	0.48
**JAG1**	28.01	±	0.87	**ITGAV**	28.80	±	1.21
**KDR**	28.02	±	1.05	**VEGFC**	28.81	±	1.20
**EGF**	28.04	±	1.00	**F3**	28.86	±	1.30
**ANG**	28.07	±	0.73	**NRP2**	28.91	±	0.42
**FGF1**	28.09	±	0.70	**FGFR3**	28.95	±	0.32
**HGF**	28.10	±	0.78	**CTGF**	28.98	±	0.79
**IFNA1**	28.11	±	1.06	**S1PR1**	29.06	±	0.60
**EPHB4**	28.11	±	0.33	**NOS3**	29.09	±	0.68
**FGF2**	28.13	±	1.51	**TGFBR1**	29.16	±	0.92
**SERPINF1**	28.15	±	0.70	**MMP14**	29.36	±	0.54
**MMP9**	28.16	±	0.54	**ID1**	29.52	±	0.64
**CCL11**	28.23	±	0.95	**TYMP**	29.56	±	1.01
**THBS2**	28.27	±	0.82	**LECT1**	29.57	±	0.87
**PDGFA**	28.34	±	0.41	**PECAM1**	29.67	±	0.65
**CXCL5**	28.36	±	1.21	**FIGF**	30.04	±	1.39
**TIMP3**	28.39	±	0.82	**ERBB2**	30.11	±	0.83
**BAI1**	28.41	±	0.46	**CCL2**	31.98	±	0.43
**ANGPTL4**	28.42	±	0.44	**TIMP1**	32.14	±	0.21
**PTGS1**	28.44	±	0.62	**MDK**	33.67	±	1.07

Proteins	*Mean Concentration*		
**IGF-I**	24000.01	±	2000.01
**TIMP-1**	10619.82	±	1934.62
**TGFB1**	8042.33	±	314.33
**Leptin**	5066.71	±	472.61
**ANG-1**	5022.84	±	896.14
**Angiostatin**	4433.35	±	321.55
**TIMP-2**	3148.77	±	221.67
**ANGPTL4**	2666.78	±	230.98
**MMP-9**	1851.36	±	30.26
**MMP-1**	1763.12	±	273.99
**Angiogenin**	1600.01	±	264.69
**Tie-1**	1562.04	±	410.55
**PECAM-1**	1269.87	±	375.74
**AgRP**	1126.88	±	264.94
**Follistatin**	1105.69	±	242.61
**uPAR**	1040.59	±	142.96
**TNFa**	963.24	±	103.56
**Activin A**	942.37	±	169.91
**FGF-4**	686.34	±	128.72
**ENA-78**	679.94	±	19.95
**LIF**	600.05	±	50.04
**G-CSF**	545.06	±	64.53
**TGFb3**	540.06	±	97.37
**Tie-2**	325.62	±	14.95
**MCP-4**	308.51	±	34.98
**RANTES**	300.02	±	50.03
**TPO**	293.54	±	73.61
**IL-12p40**	244.95	±	30.02
**IL-17**	235.76	±	79.14
**TNFb**	212.03	±	11.85
**VEGF R3**	211.12	±	41.00
**GRO**	197.93	±	12.30
**HGF**	195.93	±	19.81
**IL-1a**	184.91	±	0.71
**VEGF**	178.01	±	27.74
**VEGF-D**	159.92	±	9.07
**MCP-3**	126.60	±	24.45
**VEGF R2**	114.90	±	8.72
**IL-2**	104.70	±	29.33
**ANG-2**	93.11	±	12.66
**IFNg**	79.77	±	8.95
**TGFa**	51.18	±	5.28
**IL-8**	50.05	±	3.57
**PIGF**	49.63	±	0.88
**PDGF-BB**	46.70	±	8.27
**CXCL16**	39.71	±	5.47
**IL-6**	38.52	±	2.04
**IL-1b**	36.64	±	4.85
**IP-10**	32.65	±	1.76
**I-TAC**	28.56	±	1.09
**bFGF**	28.02	±	2.15
**MCP-2**	24.71	±	3.22
**MCP-1**	22.74	±	9.41
**IL-4**	18.05	±	5.53
**GM-CSF**	17.88	±	3.43
**I-309**	15.99	±	1.74
**IL-12p70**	11.98	±	0.85
**IL-10**	8.90	±	1.62
**HB-EGF**	8.41	±	0.70
**EGF**	2.02	±	0.64

Table S2. sEV cargo analysis. mRNA and protein quantification of e-sEV. Data are expressed as mean Ct ± SD, normalized to housekeeping genes (B2M, HPRT1, RPLP0, GAPDH, ACTB) mean Ct value (n= 4). Protein quantification values are reported as pg/ml ± SD (n= 4).

Supplementary FIGURE S2

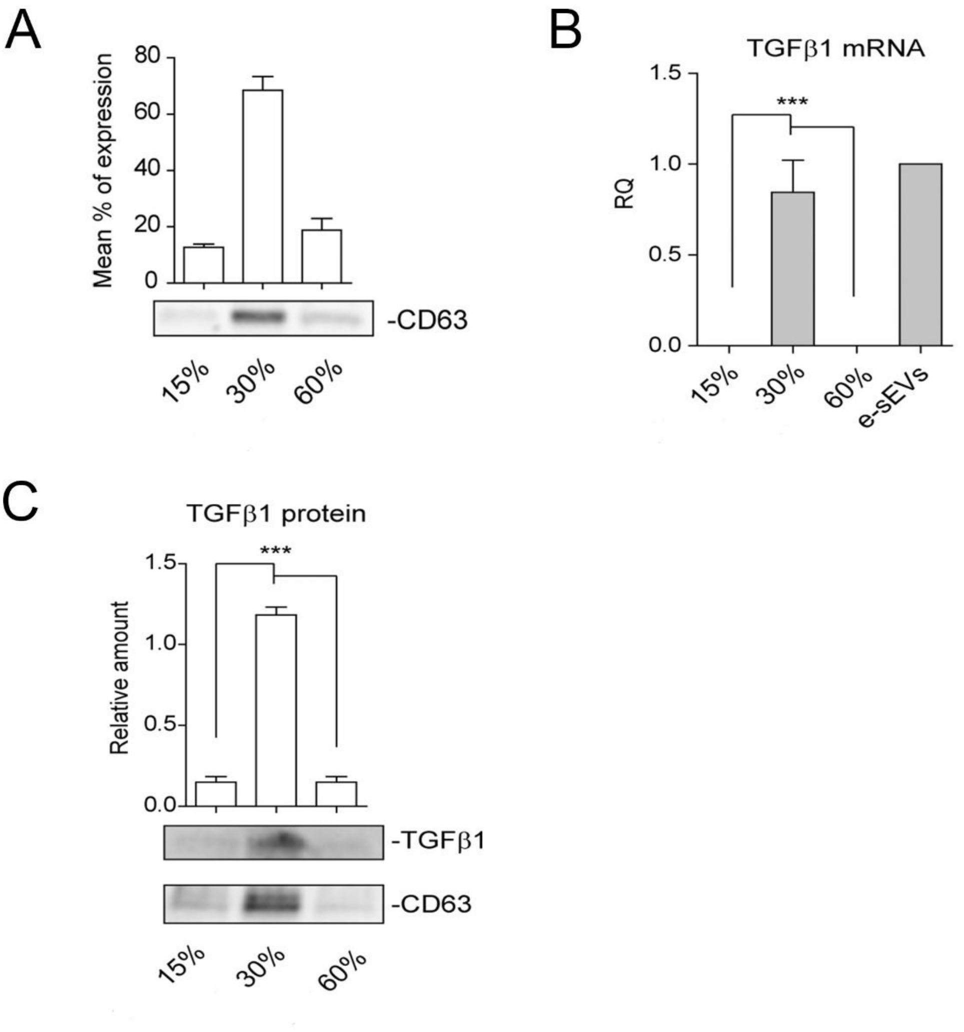

Figure S2. Analysis of TGFβ1 mRNA and protein content in e-sEVs isolated by floating density gradient. (A) Representative expression of the CD63 exosomal marker of e-sEVs isolated by floating density gradient on OptiPrep as described in Methods and subjected to Western blot analysis for CD63 exosomal marker. Mean of 3 different samples ± SEM (****p*<0.001 30% fraction vs 15% and 60% fraction). (B) e-sEVs isolated as above were analyzed for TGF□1 mRNA. e-sEVs isolated with ultracentrifugation were used as the control (****p*<0.001 30% fraction vs 15% and 60% fraction) (n=3). (C) e-sEVs isolated as above were lysed and analyzed by Western Blot for TGF□1 content. Protein levels were normalized to CD63 (****p*<0.001 30% fraction vs 15% and 60% fraction) (n=3).

Supplementary TABLE S3

Gene symbol	*e-sEV treatment (fold change)*			*Gene symbol*	*e-sEV treatment (fold change)*		
Upregulated genes				***Downregulated genes***			
**PTGS1**	8.51	±	1.45	**FIGF**	0.01	±	0.09
**ID1**	4.94	±	0.28	**TGFBR1**	0.12	±	0.59
**THBS1**	4.73	±	0.55	**HPSE**	0.14	±	0.09
**SERPINE1**	4.45	±	1.28	**TIMP3**	0.18	±	0.88
**FN1**	4.21	±	1.38	**IL8**	0.33	±	0.65
**SERPINF1**	3.53	±	1.22	**TGFB2**	0.40	±	0.82
**ERBB2**	3.22	±	1.27	**CXCL1**	0.43	±	0.72
**TIE1**	3.09	±	0.99	**ANGPT1**	0.45	±	0.54
**IFNG**	3.09	±	0.47	**HGF**	0.46	±	0.85
**CXCL9**	3.07	±	1.53	**ANGPTL4**	0.48	±	0.15
**TGFB1**	3.06	±	0.49	**FGFR3**	0.66	±	0.37
**THBS2**	2.68	±	0.07	**CXCL10**	0.78	±	0.71
**KDR**	2.52	±	1.57	**SPHK1**	0.98	±	0.16
**PECAM1**	2.50	±	0.34	**PF4**	1.05	±	0.63
**PROK2**	2.48	±	1.68	**IGF1**	1.11	±	0.58
**MMP14**	2.35	±	0.95	**F3**	1.14	±	0.62
**COL4A3**	2.34	±	1.99	**COL18A1**	1.57	±	0.84
**ANPEP**	2.32	±	1.96	**TNF**	3.72	±	0.30
**TIMP1**	2.24	±	0.30	**CXCL6**	5.80	±	0.08
**ENG**	2.19	±	0.58	**BAI1**	19.83	±	0.45
**MMP9**	2.12	±	0.23	**TYMP**	26.03	±	0.01
**IL1B**	1.90	±	1.04				
**NOS3**	1.85	±	2.42				
**HIF1A**	1.57	±	1.12				
**NRP1**	1.49	±	0.53				
**CCL11**	1.45	±	0.36				
**EFNA1**	1.33	±	0.65				
**EFNB2**	1.30	±	1.40				
**CTGF**	1.20	±	0.15				
**S1PR1**	1.16	±	0.29				
**CCL2**	1.12	±	0.62				
**NRP2**	1.12	±	0.07				
**MMP2**	1.09	±	0.04				
**PGF**	1.05	±	0.41				
**FLT1**	1.01	±	1.84				
**IFNA1**	0.96	±	0.48				
**ITGB3**	0.92	±	1.18				
**EPHB4**	0.83	±	0.17				
**TIMP2**	0.73	±	1.49				
**VEGFB**	0.72	±	1.45				
**AKT1**	0.71	±	0.90				
**CDH5**	0.68	±	0.87				
**ITGAV**	0.64	±	1.97				
**VEGFA**	0.62	±	0.61				
**NOTCH4**	0.61	±	2.04				
**MDK**	0.53	±	2.03				
**FGF2**	0.41	±	0.44				
**ANGPT2**	0.41	±	0.72				
**PLAU**	0.39	±	0.14				
**PLG**	0.38	±	0.93				
**IL6**	0.37	±	0.49				
**JAG1**	0.26	±	0.79				
**CXCL5**	0.26	±	0.58				
**LEP**	0.21	±	0.02				
**TGFA**	0.21	±	0.70				
**FGF1**	0.20	±	0.69				
**ANG**	0.17	±	0.43				
**EGF**	0.17	±	0.56				
**PDGFA**	0.16	±	0.31				
**EDN1**	0.14	±	0.27				
**VEGFC**	0.13	±	0.93				
**LECT1**	0.06	±	0.28				
**TEK**	0.06	±	0.15				

Table S3. Gene pattern expression after e-sEV treatment. Fold increase/decrease expression of 84 mRNA in ECs treated with e-sEVs. Untreated ECs were used for comparison. Data reported are the average values of four e-sEV (samples # 12, 14, 15, 17) ±SD, normalized to five housekeeping genes (B2M, HPRT1, RPLP0, GAPDH, ACTB) as described in Methods.

Supplementary TABLE S4

Gene symbol	*i-sEV treatment (fold change)*			*Gene symbol*	*i-sEV treatment (fold-change)*		
Upregulated genes				*Downregulated genes*			
**VEGFC**	48.66	±	1.49	**CXCL6**	0.16	±	0.14
**PF4**	42.12	±	0.88	**ANGPT2**	0.44	±	0.51
**PLG**	22.44	±	0.45	**TYMP**	0.55	±	1.77
**TNF**	15.74	±	2.28	**TGFBR1**	0.67	±	0.56
**PROK2**	15.73	±	1.38	**PTGS1**	0.83	±	1.23
**CCL11**	11.50	±	1.26	**ERBB2**	1.00	±	0.34
**IFNG**	10.55	±	0.97	**ITGB3**	1.35	±	1.48
**CXCL9**	8.22	±	1.29	**TIMP3**	1.45	±	1.78
**IFNA1**	7.88	±	0.87	**CCL2**	1.46	±	0.85
**COL4A3**	6.08	±	1.44	**IL6**	1.46	±	0.65
**IGF1**	5.22	±	0.56	**FN1**	1.49	±	1.00
**HGF**	4.40	±	0.17	**ANGPTL4**	1.50	±	2.01
**THBS2**	4.11	±	1.77	**SERPINE1**	1.56	±	0.66
**FGF1**	3.80	±	0.44	**COL18A1**	1.56	±	2.14
**CXCL10**	3.17	±	0.68	**MMP14**	1.56	±	0.56
**EGF**	2.36	±	0.35	**PECAM1**	1.59	±	0.78
**F3**	2.02	±	2.39	**VEGFA**	1.88	±	0.64
**ANG**	1.89	±	1.46	**VEGFB**	1.89	±	0.33
**ANGPT1**	1.89	±	0.60	**TEK**	1.92	±	0.45
**FIGF**	1.77	±	0.48	**CDH5**	2.00	±	0.70
**CXCL5**	1.66	±	0.25	**ENG**	2.02	±	0.85
**IL1B**	1.57	±	1.15	**EPHB4**	2.20	±	2.33
**MMP9**	1.48	±	2.33	**PGF**	2.24	±	0.19
**FGFR3**	1.26	±	1.23	**CXCL1**	2.35	±	0.14
**TGFB2**	1.15	±	0.63	**MMP2**	2.41	±	0.58
**LEP**	0.86	±	0.45	**TIMP1**	2.44	±	0.95
**BAI1**	0.82	±	0.75	**TIE1**	2.44	±	0.46
**HPSE**	0.62	±	1.50	**THBS1**	2.49	±	0.90
**SERPINF1**	0.60	±	0.13	**TGFB1**	2.59	±	0.58
**TGFA**	0.56	±	0.39	**NOTCH4**	2.90	±	0.78
**FGF2**	0.30	±	0.76	**NOS3**	3.34	±	0.89
**LECT1**	0.11	±	1.55	**EDN1**	3.38	±	0.90
				**PDGFA**	3.48	±	0.77
				**NRP2**	3.89	±	0.70
				**PLAU**	4.44	±	0.13
				**JAG1**	4.52	±	0.23
				**ID1**	4.88	±	0.56
				**ANPEP**	4.99	±	0.58
				**FLT1**	5.30	±	0.22
				**HIF1A**	5.43	±	0.40
				**EFNA1**	5.79	±	0.24
				**KDR**	6.02	±	0.33
				**CTGF**	6.44	±	0.53
				**MDK**	6.53	±	0.24
				**EFNB2**	6.89	±	0.65
				**IL8**	6.99	±	0.45
				**SPHK1**	7.35	±	0.44
				**ITGAV**	7.99	±	0.31
				**TIMP2**	8.13	±	0.24
				**AKT1**	8.25	±	0.19
				**S1PR1**	8.89	±	0.68
				**NRP1**	31.08	±	0.35

Table S4. Gene pattern expression after i-sEV treatment. Fold increase/decrease expression of 84 mRNA analyzed in ECs treated with i-sEVs. Untreated ECs were used for comparison. Data reported are the average values of 4 i-sEV (samples # 11, 13, 16, 18) ±SD, normalized to five housekeeping genes (B2M, HPRT1, RPLP0, GAPDH, ACTB) as described in Methods.

Supplementary TABLE S5

Up-regulated genes after e-sEV treatment/ up-regulated after i-sEV treatment	*Up-regulated genes after e-sEV treatment/ down-regulated after i-sEV treatment*	*Down-regulated genes after e-sEV treatment/ up-regulated after i-sEV treatment*	*Down-regulated genes after e-sEV treatment/ down-regulated after i-sEV treatment*
SERPINF1	ERBB2	FIGF	TGFBR1
IFNG	IL6	HPSE	TIMP3
CXCL9	PTGS1	TGFB2	IL8
THBS2	PECAM1	ANGPT1	CXCL1
PROK2	VEGFA	HGF	ANGPTL4
COL4A3	ANGPT2	FGFR3	SPHK1
MMP9	FN1	CXCL10	COL18A1
IL1B	TEK	PF4	CXCL6
CCL11	ITGB3	IGF1	TYMP
IFNA1	VEGFB	F3	
FGF2	SERPINE1	TNF	
PLG	THBS1	BAI1	
CXCL5	CDH5		
LEP	ENG		
TGFA	CCL2		
FGF1	MMP14		
ANG	EPHB4		
EGF	PGF		
VEGFC	TGFB1		
LECT1	TIMP1		
	MMP2		
	TIE1		
	NOTCH4		
	PDGFA		
	NRP2		
	EDN1		
	NOS3		
	PLAU		
	JAG1		
	ID1		
	ANPEP		
	FLT1		
	HIF1A		
	EFNA1		
	KDR		
	MDK		
	CTGF		
	EFNB2		
	AKT1		
	ITGAV		
	TIMP2		
	S1PR1		
	NRP1		

Table S5. List of genes regulated upon e-sEV and i-sEV treatment. The Table summarizes the list of genes that are up-regulated and down-regulated upon e-sEV and i-sEV treatment.

Supplementary TABLE S6

Gene symbol	*VEGF (fold change)*			*Gene symbol*	*VEGF (fold change)*		
Upregulated genes				***Downregulated genes***			
**IL6**	3.38	±	1.32	**AKT1**	0.02	±	0.02
**VEGFA**	3.36	±	0.54	**FIGF**	0.14	±	0.13
**VEGFC**	3.33	±	0.10	**CDH5**	0.17	±	0.28
**PTGS1**	3.30	±	0.34	**COL18A1**	0.21	±	1.61
**PGF**	3.21	±	0.10	**ANG**	0.21	±	0.45
**TIE1**	3.12	±	0.28	**IL8**	0.23	±	0.11
**PECAM1**	3.08	±	0.25	**TGFB1**	0.31	±	0.10
**ID1**	3.07	±	0.29	**LEP**	0.33	±	0.57
**JAG1**	3.04	±	2.32	**TGFA**	0.37	±	0.66
**NOS3**	3.01	±	0.54	**CXCL1**	0.44	±	0.59
**ITGB3**	2.83	±	1.44	**TGFB2**	0.47	±	0.04
**THBS1**	1.77	±	1.02	**IL1B**	0.55	±	0.30
**ERBB2**	1.62	±	0.35	**SPHK1**	0.56	±	0.23
**FN1**	1.56	±	0.54	**TEK**	0.65	±	0.13
**CTGF**	1.38	±	1.72	**ENG**	0.69	±	1.95
**NRP1**	1.31	±	1.04	**TIMP3**	0.74	±	0.70
**ANPEP**	1.30	±	1.53	**VEGFB**	0.75	±	0.69
**HIF1A**	1.28	±	0.29	**IGF1**	0.77	±	0.51
**TIMP2**	1.24	±	1.48	**TNF**	0.87	±	0.75
**NRP2**	1.23	±	1.22	**PLG**	0.99	±	0.25
**HPSE**	1.22	±	1.73	**ANGPTL4**	0.99	±	0.23
**EFNA1**	1.18	±	0.24	**CXCL10**	1.18	±	0.00
**PLAU**	1.15	±	1.71	**CXCL5**	1.61	±	0.54
**F3**	1.12	±	0.96	**CXCL6**	1.81	±	0.12
**FGF2**	1.12	±	0.31	**TYMP**	2.22	±	0.44
**S1PR1**	0.95	±	1.42	**ANGPT1**	2.48	±	0.41
**SERPINE1**	0.92	±	1.64	**CXCL9**	2.89	±	0.23
**COL4A3**	0.87	±	0.26	**CCL11**	4.03	±	0.28
**EDN1**	0.85	±	0.43	**THBS2**	4.38	±	0.23
**ITGAV**	0.83	±	0.01	**LECT1**	6.58	±	0.19
**CCL2**	0.75	±	1.33	**BAI1**	7.00	±	1.27
**SERPINF1**	0.68	±	1.60	**MMP9**	7.85	±	0.16
**EPHB4**	0.63	±	1.07	**FGFR3**	9.00	±	0.23
**TIMP1**	0.63	±	0.43	**IFNG**	9.26	±	0.14
**FLT1**	0.58	±	0.64	**HGF**	14.87	±	0.09
**EFNB2**	0.56	±	1.68	**EGF**	16.86	±	0.01
**MMP2**	0.50	±	0.38				
**FGF1**	0.46	±	1.72				
**ANGPT2**	0.44	±	0.09				
**KDR**	0.42	±	1.19				
**TGFBR1**	0.42	±	0.32				
**MMP14**	0.42	±	0.00				
**PF4**	0.34	±	0.83				
**IFNA1**	0.30	±	0.69				
**MDK**	0.24	±	1.31				
**PDGFA**	0.21	±	0.24				
**NOTCH4**	0.19	±	1.68				
**PROK2**	0.06	±	0.60				

Table S6. List of genes regulated upon VEGF treatment. Fold increase/decrease of 84 mRNA in ECs treated with VEGF (10 ng/ml). Untreated ECs were used as control. Data reported are the average values of four VEGF treatments ±SD, normalized to five housekeeping genes (B2M, HPRT1, RPLP0, GAPDH, ACTB) as described in Methods.

Supplementary FIGURE S3

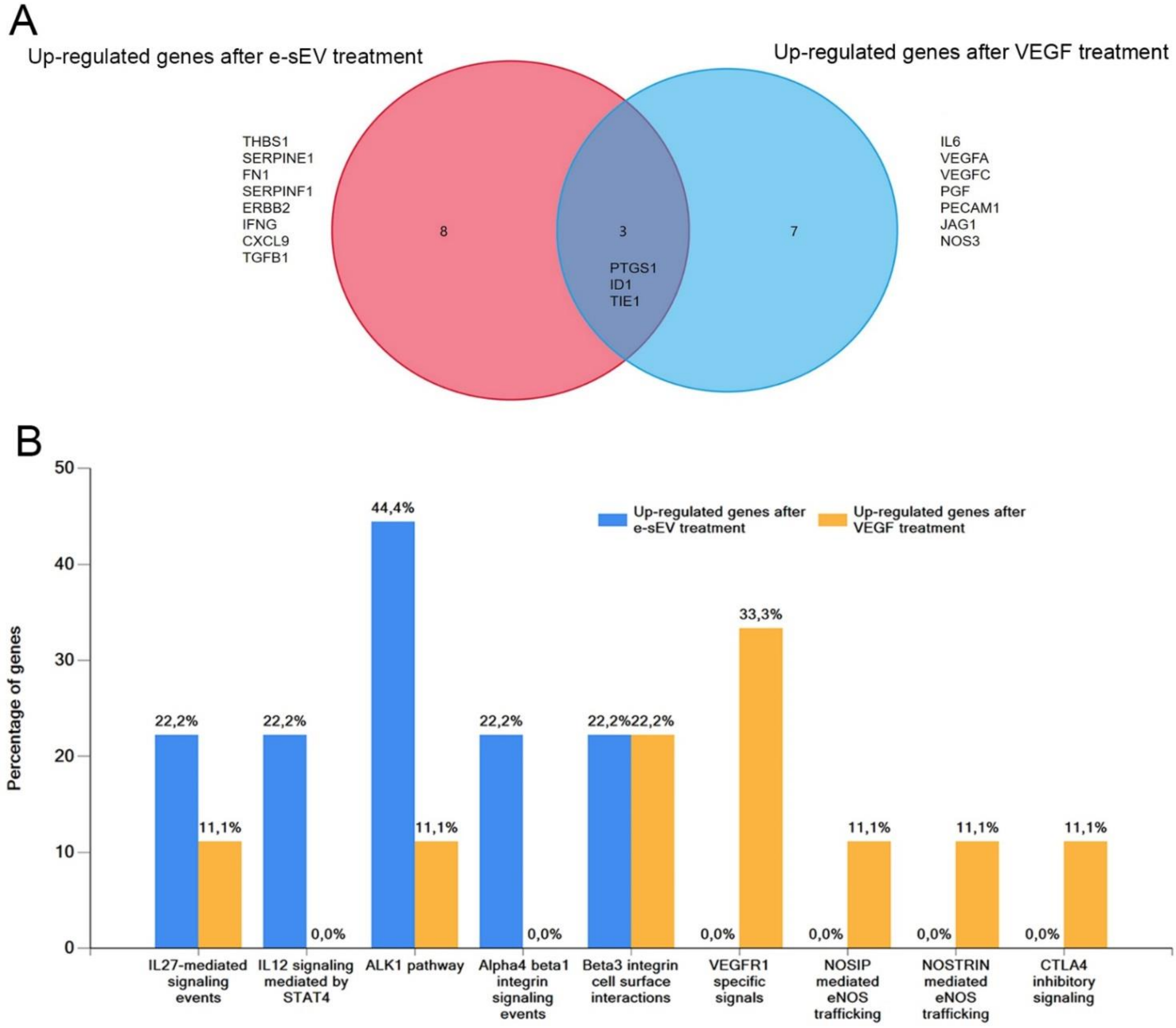

Figure S3. Comparison between genes regulated upon e-sEV and VEGF treatment. (**A**) The Venn diagram reports the lists of selected up-regulated genes (fold increase ≥ 3) after e-sEV and VEGF treatment. PTGS1, ID1, TIE1 were shared. (**B**) The histogram represents the GO signaling pathways related to genes listed in the Venn diagram. Columns represent the % of gene clusters involved in each signaling pathway.

Supplementary FIGURE S4

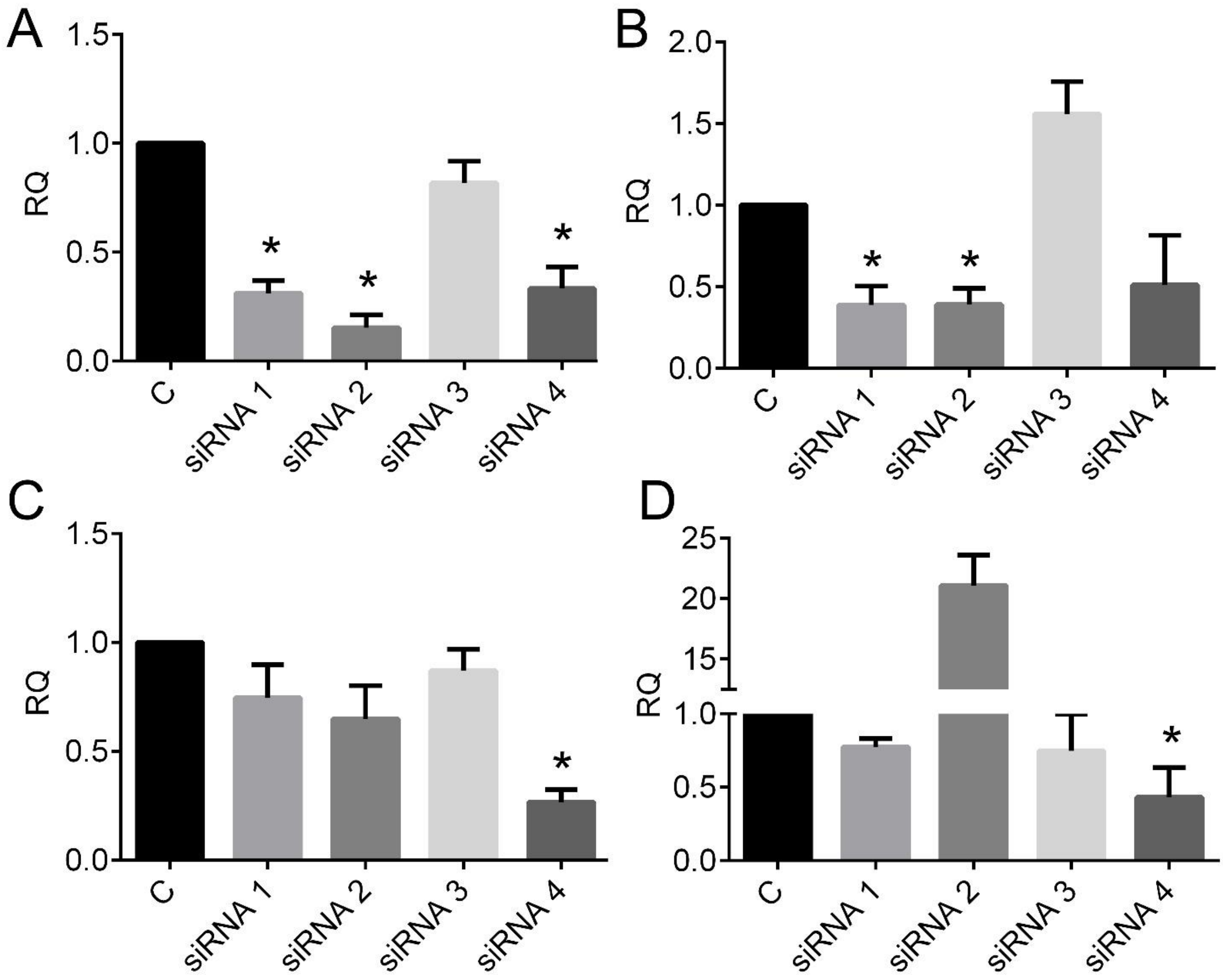

Figure S4. Analysis of siRNA for SMAD1/5 on ECs. RT-PCR on ECs transfected with duplex siRNAs or 4 different siRNA vectors for SMAD1 and SMAD5 at different time points (48 and 72 hours). Histograms report the SMAD1 or SMAD5 RQ values of transfected cells with respect to ECs transfected with duplex siRNAs (C). Efficient siRNAs were selected for ChIP analysis: siRNA 2 SMAD1 48h and siRNA 4 SMAD5 48 h. **p < 0.01 C vs siRNA (n=4); (One-way ANOVA followed by Tukey's post hoc test).
